# Learning and Motivation State Fluctuations from Motoric and Neurophysiologic Metrics during a Somatosensory Task in Mice

**DOI:** 10.1523/ENEURO.0417-25.2026

**Published:** 2026-05-26

**Authors:** Lezio S. Bueno-Junior, Anjesh Ghimire, Mingxin Ding, Brendon O. Watson

**Affiliations:** ^1^Department of Psychiatry, University of Michigan Medical School, Ann Arbor, Michigan 48109; ^2^Department of Psychology, Brandeis University, Waltham, Massachusetts 02453

**Keywords:** eye movements, laminar neocortical activity, motivation fluctuations, performance variations, wheel running, whisker stimulation

## Abstract

Animal learning can be analyzed on two timescales: task acquisition across training sessions and motivation fluctuations within training sessions. How do variations in motor and neurophysiologic activity relate to task performance over these timescales? Here, this question was examined in head-fixed mice performing a whisker-based sensory discrimination task. Male mice were trained for 12–14 daily sessions on a go/no-go task, each lasting ∼1 h to capture spontaneous performance fluctuations over minutes. Simultaneous to task performance, “nonperformance variables” were tracked, including wheel running, pupil size, eyelid aperture, and sensory cortical activity. First, motivation states were defined based on performance tendencies over minutes, leading to three state categories: persistent, disengaged, or attentive. Nonperformance variables were found to predict these states independent of task correctness. Then, when further parsing these states by the go/no-go outcomes of hit, miss, false alarm, or correct rejection, learning-like changes were detected in wheel running, eye movements, and brain activity. Thus, learning over days and motivation fluctuations over minutes form a continuum, as evidenced by changes in motor and physiologic activity variables not directly controlled by task contingencies, even during periods of suboptimal performance in well-trained subjects. These findings improve the understanding of performance variations and implicit learning, in addition to contributing a framework for the analysis of task performance indirectly from motor and neurophysiologic activity.

## Significance Statement

Task performance is typically measured by correctness percentages over daily training sessions but can also correlate with motivation state fluctuations within each session. Thus, while aggregating correctness percentages per session may reveal a learning curve, accounting for within-session state fluctuations can reveal variability in that curve, even among well-trained subjects. In this head-fixed mouse study, task acquisition across sessions and motivation fluctuations within sessions were categorized into three states: persistent, disengaged, or attentive. Subsequently, metrics not directly controlled by the task, including locomotion, pupil dilation, eyelid aperture, and brain activity, were found to predict both learning and state changes. Therefore, task performance can be tracked more comprehensively from brain and body activity metrics, adding nuance to correctness percentages.

## Introduction

Behavioral performance in learned tasks can be measured relative to the amount of training or experience. This is often referred to as the learning curve: the psychological concept that task performance improves with training until reaching a plateau, after which point performance stabilizes or fluctuates despite further training (Thorndike, 1905; Skinner, 1936; Moore and Kuchibhotla, 2022). Task performance can also be measured relative to motivation levels. This is often characterized by an inverted-U relationship between such levels and performance: either too much or too little motivation can lead to worse performance than what is achieved at intermediate motivation (Yerkes and Dodson, 1908; Kim and Kim, 2023).

Arousal and motivation states are known to fluctuate at timescales shorter than a training session (e.g., minutes to tens of minutes), causing performance inconsistencies even in well-trained subjects (Yin et al., 2025). Thus, learning curves across sessions and state-dependent performance fluctuations within sessions could be manifestations of the same continuum. These shorter-timescale (or intrasession) performance variations were traditionally dismissed as noise but have been increasingly emphasized over the last decade due to technical developments in behavioral and physiological monitoring, combined with advances in modeling and analytics (Boogert et al., 2018). While these developments have accelerated research on the learning-state continuum, some questions remain open for further exploration: do states follow a typical progression within a training session? Is that progression consistent within and across subjects? Is learning also observable during poor performance? What are the nonperformance markers of these performance changes?

Here these questions were examined in head-fixed mice performing a whisker-based go/no-go task. Go/no-go decisions and response rates (licking) were tracked as measurements of motivation and performance tendencies over minutes. Simultaneously, “nonperformance variables” were tracked, including prestimulus licking, wheel running, pupil size, eyelid aperture, and laminar electrophysiology from the sensory cortex, none of which directly controlled by the go/no-go contingencies. According to the results, nonperformance variables can predict motivation states on an individual subject basis, in addition to changing over the days of training, including in periods outside optimal performance. These findings are consistent with previous studies (Kuchibhotla et al., 2019; Matteucci et al., 2022; Hulsey et al., 2024; Yin et al., 2025) by corroborating that (1) acquisition and expression of learned behaviors are not fully correlated; (2) task acquisition is accompanied by the formation of collateral, uninstructed behaviors; and (3) both motivation and learning modulate stimulus responses in the sensory cortex. This study builds upon the literature by contributing a unified view of the learning-motivation continuum, which appears to be malleable, individualized, and quantifiable from the perspective of nonperformance metrics.

## Materials and Methods

### Subjects

Five adult male mice (C57BL/6) were trained on the behavioral task and their data analyzed. The mice were housed with standard bedding under a 12 h light/dark cycle (lights turning on at 6 A.M.). Upon arrival at the vivarium at 9–10 weeks of age, the mice were kept in groups of 2–3 per cage. Surgeries were performed when the mice were 11–13 weeks old, after which they were housed individually to prevent damage to headcap implants by cage mates (see details on surgeries below). Experiments were conducted between 7 A.M. and 12 P.M. (lights-on timing seasonally varied between 6 and 7 A.M.). Mice had *ad libitum* access to food throughout the experiments and restricted access to water specifically during behavioral training (see below, Head fixation, wheel running, and licking). All procedures adhered to the National Institutes of Health Guide for the Care and Use of Laboratory Animals and were approved by the Institutional Animal Care and Use Committee of the University of Michigan (protocol 00011423).

### Head fixation, wheel running, and licking

**Figure 1.**
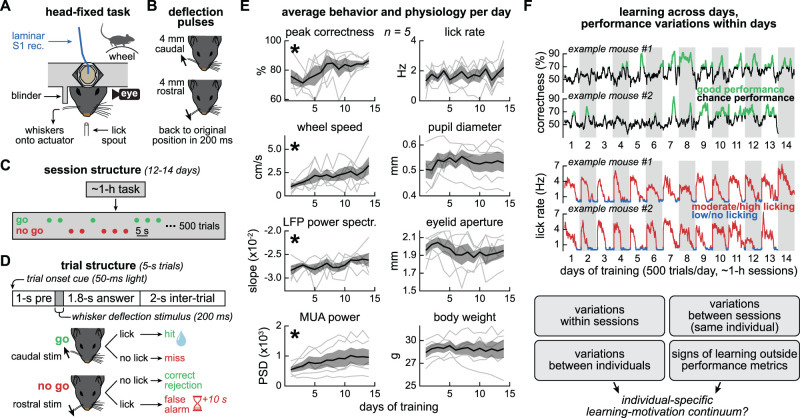
Experimental design, summary of dataset, and study objects. ***A***, Mice were implanted with a chronic electrophysiology headcap and trained on a head-fixed task involving whisker stimulation, licking, and water rewards. Wheel, eye, and brain activity from the S1 cortex (Extended Data Fig. 1-1) were recorded concurrently. ***B***, Tactile stimuli consisted of caudal or rostral deflection pulses (200 ms) delivered to somatotopically identified whisker pairs. ***C***, Training sessions were conducted daily for 12–14 d, each session comprising a pseudorandom sequence of 500 go/no-go trials. ***D***, Each trial lasted 5 s, divided into prestimulus, the stimulus itself, answer period, and intertrial period—the latter was extended to 10 s following false alarms. Go and no-go trials were signaled by caudal and rostral whisker deflections, respectively. Water rewards or false-alarm extensions were triggered just once per trial, upon detection of the first lick anytime within the answer period. Further licking in that trial, whether during or after water consumption, did not trigger additional water delivery. ***E***, Summary of the behavior and physiology dataset across mice (*n* = 5), showing daily averages with or without learning-related trends over training. ***F***, Correctness and lick rate data from two representative mice, one data point per trial. In this illustration, trials were concatenated across days (500 trials/day) to show variations within and between sessions, as well as between mice. These variations were analyzed as manifestations of individual “learning-motivation continua.” See also Extended Data Figures 1-2 and 2-1.

Awake mice were head-fixed using 3D-printed parts based on the RIVETS head fixation system (Osborne and Dudman, 2014), including a head fixation ring cemented within an implanted headcap (Fig. 1*A*), similar to a previous study (Bueno-Junior et al., 2023). See surgery procedures below for details on the headcap implant. While head-fixed, the mice were allowed to walk on a 3D-printed wheel (13 cm diameter; designed in SolidWorks) attached to a miniature absolute magnetic shaft encoder (US Digital) for recording locomotion (Fig. 1*A*).

During head fixation, whether during task acclimatization or task execution, water drops were delivered using a lick spout (Fig. 1*A*) controlled by a solenoid valve driver (Bpod, Sanworks), depending on task responses. Specifically, a water container (50 ml beaker) was placed on a shelf 40 cm above the recording setup, creating pressure to transmit water down through two segments of plastic tubing (0.8 mm inner diameter) connected by a solenoid valve. At each reward, the valve was opened for a discrete interval (50 ms), producing a small water drop (∼1 mm) on the lick spout (metal tube, 0.8 mm inner diameter) at the distal end of the plastic tubing. The water drop remained adhered to the spout tip due to capillarity, until consumed by the mouse. All licks, rewarded or not, were timestamped as infrared beam breaks, produced by an infrared emitter and phototransistor pair near the lick spout (0.4 cm each side). A white LED located 0.8 cm from the lick spout signaled the onset of each trial. Lick spout, infrared emitter/phototransistor, and white LED were all enclosed within a miniature 3D-printed casing (Bpod, Sanworks).

Starting a week before training, mice received restricted access to water in the vivarium (15 min daily), which continued throughout the training. Two days before training, mice were acclimated to both head fixation and lick-based water delivery in daily sessions, ∼1 h each. Water drops were administered using a graphical user interface (Bpod Console, Sanworks) during acclimation; licking behavior was not recorded in these sessions due to their experimenter-controlled nature. During both acclimation and subsequent training, free water was provided in the vivarium after each training session, and all mice consistently displayed a high motivation to drink. This suggests that satiation alone could not explain the spontaneous performance variations reported here, as discussed later. Moreover, water restriction was fixed across mice without adjustments by body weight, allowing again for spontaneous performance variations.

### Whisker stimulation

Using SolidWorks and 3D printing, a blinder was included in the head fixation system, near the right eye, preventing the animal from seeing the whisker stimulator (Fig. 1*A*). The whisker stimulator consisted of a custom 3D-printed pole (also designed in SolidWorks), movable by a linear actuator (Zaber, NA series) under the control of voltage commands from Bpod State Machine (Sanworks). The stimulator was designed to move at the same horizontal plane of the snout, parallel to the mystacial pad, ∼5 mm away from it (Fig. 1*B*). Upon receiving voltage commands, the actuator was programmed (in C#) to move 4 mm caudally or rostrally in 100 ms and return to its starting position in another 100 ms, resulting in deflection pulses of 200 ms, similar to a previous study (Schriver et al., 2018). The whiskers moved passively with each pulse (Fig. 1*B*), as the whiskers were temporarily adhered to the stimulator using a double-sided adhesive dispenser. Active whisker palpation or high-speed whisker imaging were disregarded, as these were outside the scope of this study.

### Eye and pupil video

For eye videography, a laterally positioned camera (7 cm distance between lens and mouse) framed the left eye, contralateral to the stimulated whiskers (Fig. 1*A*). Only the left eye was tracked, which was deemed sufficient for monitoring motivation states while allowing the placement of a blinder between the right eye and the whisker stimulator (Fig. 1*A*). The hardware consisted of an infrared-sensitive 10 bit grayscale camera (FL3-U3-13S2 M, Flir) coupled with a VZM 200i lens (Edmund Optics). Illumination was achieved via infrared reflectance using an infrared LED board close to the lens and pointing toward the eye, i.e., without trans-skull infrared illumination as described previously (Yüzgeç et al., 2018; Tsunematsu et al., 2020; Sattler and Wehr, 2021). The videos were acquired via USB card (U3-PCIE2-2P01X), with frame rate (20 frames/s) and frame size (640 × 512 pixels) configured through the FlyCapture software (Flir). Videos were written into AVI files (M-JPEG codec) using the same software. The camera also generated digital timestamps (1 ms duration, one per frame) for data integration. All components were assembled using a custom optomechanical setup (Thorlabs).

### Go/no-go training

The training consisted of 12–14 daily sessions, depending on the mouse (Fig. 1*C*). Each training session lasted ∼1 h, comprising a pseudorandom sequence of 500 “go” or “no-go” trials, with no more than three consecutive repetitions of the same trial type (Fig. 1*C*). Each trial began with a trial onset light cue (a 50 ms white LED flash) and was divided into 1 s prestimulus period, 200 ms stimulus, 1.8 s answer period, and 2 s intertrial interval (Fig. 1*D*). Each stimulus consisted of a whisker deflection pulse administered just once per trial. The trial type was indicated by the direction of the whisker deflection: caudal for go and rostral for no-go (Fig. 1*D*). Based on the presence or absence of poststimulus responses, trials were further sorted into the four standard go/no-go outcomes: hit (lick in go trials, rewarded with water), miss (no lick in go trials, no outcome), correct rejection (no lick in no-go trials, no outcome), or false alarm (lick in no-go trials, punished with extended intertrial interval of 10 s; Fig. 1*D*). Water rewards or false-alarm extensions were triggered just once per trial, upon detection of the first lick anytime within the 1.8 s answer period (Fig. 1*D*). Each water reward consisted of a single drop (∼1 mm) per trial, which remained adhered to the spout tip by capillarity until consumed by the mouse, as explained previously. Further licking in that trial, whether during or after water consumption, did not trigger additional water delivery.

### Pretraining surgeries

Craniotomy surgeries were performed before all acclimatization and training sessions (i.e., before the main experiments depicted in Fig. 1*A–D*) and are now described in detail. Two surgeries were performed per animal, 1 week apart: one for intrinsic optical imaging of the primary somatosensory cortex (S1) barrel field and another for imaging-guided silicon probe implantation across S1 layers (Extended Data Fig. 1-1).

#### Surgery 1: intrinsic optical imaging

Intrinsic optical imaging procedures followed protocols established previously (Aronoff and Petersen, 2007; Knutsen et al., 2016), with adaptations. Mice were anesthetized with isoflurane (1–1.5% v/v in oxygen), secured in a stereotaxic frame, and kept at 37°C body temperature by using a heating pad. All whiskers on the right side were trimmed except for six of them: C1-2, D1-2, and E1-2, which were reserved for subsequent stimulation. Following a midline incision of the scalp, a square thinned-skull window was created above the left (contralateral) S1 barrel field area using a drill. The size and position of the skull window were based on coordinates for barrels C1-2, D1-2, and E1-2 (Knutsen et al., 2016), corresponding to the spared whiskers. The skull window was kept wet with saline and covered with a glass coverslip for reversible skull transparency.

Images of the skull window were captured using a 12 bit grayscale camera (MV1-D1024E-G2, PhotonFocus) connected to a VZM 200i lens (Edmund Optics), positioned at a 30° angle relative to the sagittal plane to align with the curvature of the S1 cortex. For illumination, a high-power LED system (Roithner LaserTechnik) was used, alternating between red light (630 nm) for functional imaging of the brain surface and green light (525 nm) for a snapshot of blood vessels on the brain surface—the latter was used as anatomical reference for functional imaging. For whisker stimulation, 10 Hz rostral–caudal vibrations were administered to one whisker pair at a time (C1-2, D1-2, E1-2) by placing whisker pairs into a glass micropipette attached to a piezoelectric actuator (Piezo.com). This apparatus was mounted on a custom-built optomechanical setup (Thorlabs). Both the camera and piezoelectric actuator were synchronously triggered by a Bpod State Machine (Sanworks).

For each whisker pair, red-illuminated frames were collected at 4 Hz during a 4 s baseline, 4 s whisker stimulation, and 4 s intertrial interval, for a total of 40 trials. Using the Image Acquisition Toolbox in MATLAB (MathWorks), stimulation frames (stim) and baseline frames (no-stim) were normalized against the average baseline image of that trial. The normalized frames were then averaged across trials, resulting in images with and without intrinsic signal (one stim/no-stim image pair per whisker pair). Brain surface signals were smoothed, binarized (pixels beyond 1.5–2 standard deviations within parenchymal areas were classified as signal), and merged onto the blood vessel snapshots mentioned above, creating mouse-specific maps to guide future implantation (Extended Data Fig. 1-1).

After imaging, the glass coverslip was removed and four microscrews were inserted into the bone surrounding the thinned-skull window. An additional burr hole was made over the contralateral cerebellum to insert a connector pin (22 AWG) for future electrical grounding. Subsequently, a 3D-printed head fixation ring (Osborne and Dudman, 2014) was positioned on the skull, around the microscrews and ground pin. These were all cemented together using adhesive cement (C&B Metabond, Parkell) except for the thinned-skull window, which was covered with silicone sealant (Kwik-Cast) for later removal in Surgery 2. An analgesic and anti-inflammatory treatment (carprofen, 5 mg/kg, s.c.) was administered 1 h before concluding the surgery, and a 1 week recovery period was allowed.

#### Surgery 2: imaging-guided silicon probe implantation

For Surgery 2, 1 week later, mice were once again anesthetized and secured to the stereotaxic frame. The stereomicroscope was set up to visualize the thinned-skull window prepared in Surgery 1. Utilizing a micromanipulator (Sensapex), a silicon probe (one linear shank with 64 electrode sites along its edge, spaced 20 µm apart; NeuroNexus) was lowered toward the location of the skull window corresponding to the strongest intrinsic imaging signal (Extended Data Fig. 1-1) at 30° relative to the sagittal plane, without touching the skull. The silicon probe was kept plugged into an amplifier/digitizer headstage system (Intan) to collect anesthetized electrophysiology during implantation. See the next subsection for details on electrophysiology.

The silicon probe was then retracted for removal of the thinned skull, and reference wires from the probe connector package were plugged into the ground pin. The probe was then lowered back toward the brain while recording, until touching the dura mater, as indicated by a cross-channel voltage artifact. The dorsal–ventral reference was set to zero upon touching the dura mater. The probe was further lowered into the brain, with real-time monitoring using an electrophysiology viewer (NeuroScope). Transitions from electrical noise to brain signal were visualized on a channel-by-channel basis, as the silicon probe was lowered. The trajectory was halted when the superficial channels began to display brain signals.

Once the implantation was complete, the silicon probe shank, its connector package, and the ground wiring were cemented (Unifast Trad) on top of the preexisting headcap base prepared during Surgery 1. The implantation procedure was concluded by disconnecting the headstage from the silicon probe connector and releasing the mouse. Analgesic anti-inflammatory treatment (carprofen, 5 mg/kg, s.c.) was administered again 1 h before completing the surgery, followed by an additional week for postsurgical recovery.

### Electrophysiology, data integration, and somatotopy check

Brain activity was amplified (1,000×) and digitized (20 kHz) by a 64-channel headstage (Intan) and transmitted through an SPI cable to a multichannel interface (RHD2000 system, Intan). The raw signal was preprocessed into local field potentials, LFP (450 Hz low-pass filtering, downsampled to 1,250 Hz), and multiunit activity, MUA (500 Hz high-pass filtering). For Faraday protection, the ground channel of the multichannel interface was wire-connected to a sheet of metal mesh surrounding the head fixation setup. Sources of electrical noise (e.g., infrared illuminators) were then connected to the metal mesh. In addition to recording brain activity via the silicon probe (NeuroNexus), the multichannel interface recorded wheel activity (shaft encoder, US Digital), timestamps for eye/pupil video frames (Flir camera), whisker stimulation commands, and licks (Bpod)—all at the unifying sampling rate of 20 kHz.

For further specification of whisker-barrel somatotopy, one passive whisker stimulation session was conducted per mouse, under awake head fixation but before the actual behavioral training. A randomly chosen whisker pair (C1-2, D1-2, or E1-2, untrimmed during surgery) was temporarily adhered to the stimulator and deflected caudally or rostrally in a random sequence of trials, every 5 s for 15 min. This process was then repeated for the other whisker pairs. Following the passive stimulation session, electrophysiological epochs from all channels were extracted per stimulus (50 ms before, 250 ms after stimulus onset) and averaged across stimuli for current-source density (CSD) analysis (explained below). Mean absolute CSD responses were then measured across channels and poststimulus time. The whisker pair associated with the maximum CSD response was spared for the remainder of the study to ensure extra somatotopic targeting. All other whiskers on the right side of the animal were trimmed weekly under isoflurane anesthesia. The whiskers on the left side of the animal (i.e., ipsilateral to the silicon probe implant) were not manipulated throughout the study.

### Data analysis

All data were analyzed in MATLAB (MathWorks). The subsections below indicate the data figures corresponding to each method.

#### Summary values per session

For Figure 1*E*, summary values were calculated per session and their variations across mice were illustrated with ±standard error curves. Significantly ascending curve directions (indicated by asterisks) were determined by linear correlations between the data (aggregated from all mice) and training days (Fig. 1*E*). The summary values are explained in the items below. See also Extended Data Figure 1-2 for qualitative illustrations of within-session data.

*Peak correctness*. A binary vector (length = 500 trials) was constructed per session to indicate both hits and correct rejections as “1” and other trial outcomes as “0.” The vector was smoothed with a moving mean of 50-trial window and 1-trial step size. The maximum value of the smoothed vector was then taken per session.

*Wheel speed.* Rotations from the shaft encoder were recorded by the multichannel interface into a voltage range (0–3.3 V) and rescaled to a centimeter range (0–36 cm) corresponding to the circumference of the wheel. The centimeter range was converted into an ever-ascending curve representing traveled distance (*unwrap* function) and then into a speed curve in cm/s (*diff* function). The mean speed was finally calculated per session.

*Power spectrum slope (PSS) from LFP*. One LFP epoch (1,250 Hz) was extracted per channel and per trial. These epochs were taken from the 1 s prestimulus period to minimize confounds from electromyographic activity caused by licking (see below for analysis of stimulus-evoked LFP responses). For each epoch, a power spectral density curve (PSD, *pwelch* function) was generated using the following parameters: frequency range, 1–100 Hz; 1 Hz bin size (logarithmically spaced); and window, 1 s (50% overlap). This PSD was converted to base 10 logarithm and linearly fitted. The slopes of these fits (i.e., PSS) were obtained per trial and averaged across channels. The mean PSS was then calculated per session by averaging across trials.

*MUA power*. One raw epoch (20 kHz) was extracted per channel and per trial. These epochs were taken from the 1 s prestimulus period to minimize confounds from electromyographic activity caused by licking, like the PSS analysis (see below for analysis of stimulus-evoked MUA responses). For each epoch, a PSD curve (*pwelch* function) was generated using the following parameters: frequency range, 500–5,000 Hz, 500 Hz bin size (linearly spaced), and window, 0.5 s (50% overlap). MUA band power was calculated as the sum of PSD values across frequency bins, per channel. The mean MUA power was then calculated per session by averaging across channels and trials.

*Lick rate*. The total number of licks was divided by the total duration of the session, resulting in a summary value in hertz.

*Pupil diameter and eyelid aperture*. Eye activity was tracked using DeepLabCut, a supervised keypoint tracker (Nath et al., 2019). DeepLabCut was trained to place eight keypoints on the eyelids and another eight keypoints around the pupil. The pixel coordinates of these keypoints were converted to millimeters and analyzed as irregular octagons that moved dynamically across video frames. Pupil diameter and eyelid aperture were measured as the distance (hypotenuse) between their topmost and bottommost vertices. Pupil diameter and eyelid aperture were averaged across the video frames of each trial, except punishment periods. These eye activity metrics were then averaged across trials, per session.

*Body weight*. Body weight (grams) was measured prior to each session using a scale near the head fixation setup.

#### State classification

**Figure 2.**
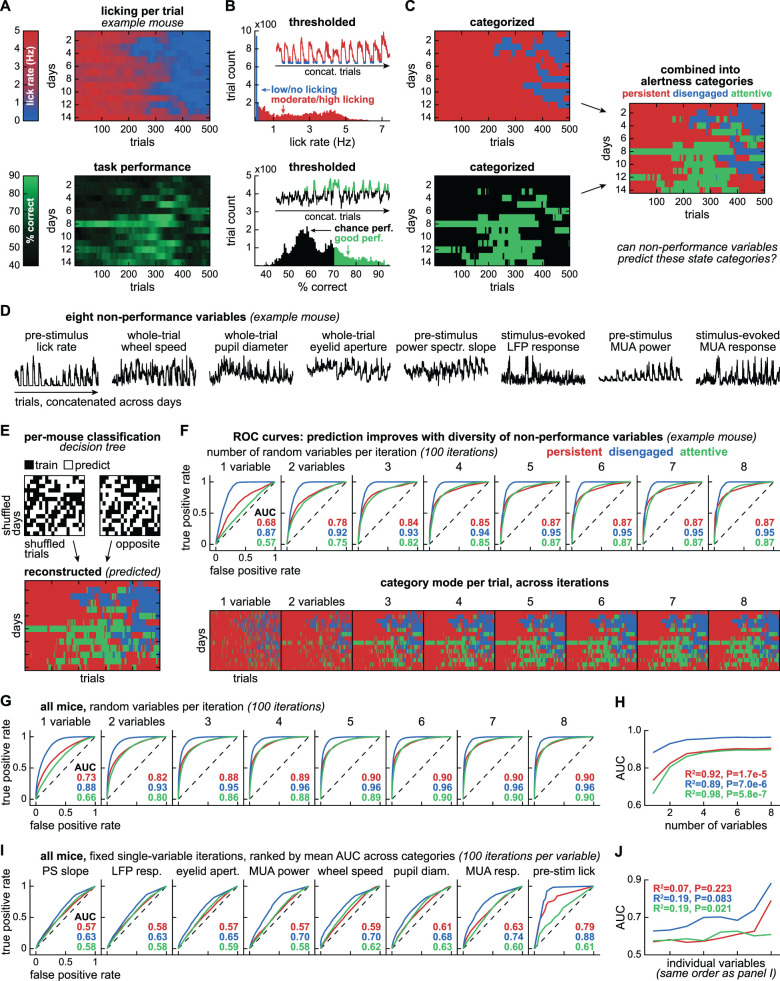
Performance and nonperformance metrics covary with learning and motivation state. ***A***, Day-by-trial matrices (14 rows, 500 columns) from a representative mouse, color-coded for whole-trial lick rates (top) and correctness percentages (bottom). Each daily session was smoothed independently with a 50-trial moving mean (1-trial steps). Lick rates typically decreased abruptly after ∼250–350 trials. High correctness tended to emerge in the middle of each session, generally after 4–6 d of training. However, these trends varied within and between mice. ***B***, Such variations were classified algorithmically by binarizing lick rates and correctness percentages (see also Fig. 1*F*). ***C***, The binary lick rate and correctness matrices were then combined into a trial classification system identifying “persistent,” “disengaged,” and “attentive” states. ***D***, Additional examples of concatenated trial curves (similar to panel ***B***), but this time from eight nonperformance variables, illustrating different levels of redundancy among the variables. ***E***, Decision-tree classifiers were trained on these nonperformance variables to recreate the “persistent,” “disengaged,” and “attentive” trial categories. As a control, the trial order was shuffled and performance variables (whole-trial lick rates and correctness percentages) were omitted from the classification inputs. ***F***, Illustrative ROC AUC analysis from the same mouse as in panels ***A***–***E***. Classification was conducted in eight groups of 100 iterations, each group with a different number of randomly chosen nonperformance variables. Cumulative true-positive and false-positive rates were then calculated per group of 100 iterations, showing that the more diverse the pool of nonperformance variables, the better the classification. ***G***, Same as panel ***F*** but from all mice. ***H***, *R*-squared correlations between the number of nonperformance variables and AUC from all mice. AUC increased steeply up to four variables and then incrementally up to eight variables, especially for “persistent” and “attentive” categories. ***I***, Same as panel ***G***, but from fixed (nonrandom) single-variable iterations, ranked by mean AUC across states. No single variable matched the classification performance obtained from multiple variables. ***J***, Same as panel ***H***, but from single-variable iterations. The *x*-axis corresponds to the ranked variables shown in panel ***I***. These data show that animal learning can be inferred from a collection of motor and physiologic variables, even if excluding correctness and response rate metrics. See also Extended Data Figures 2-1–2-5.

Each task trial was algorithmically assigned to one of three states—“persistent,” “disengaged,” “attentive”—based on a combination of licking activity and task performance thresholds specific to each mouse (Figs. 1*F*, 2*A*–*C*).

*Licking activity*. A lick rate vector (length, 500 trials) was constructed per session, each value (in hertz) representing the whole 5 s period of each trial (punishment periods excluded). These vectors were smoothed using a 50-trial moving mean to capture state variations over several trials (Fig. 2*A*, top), on timescales ranging from minutes to tens of minutes (trial-by-trial variations were outside the scope of this study). This 50-trial window was selected heuristically through visual comparison of multiple window sizes, deemphasizing trial-by-trial variability while emphasizing longer-term state fluctuations. All trials were weighted equally in each window, which progressed in one-trial steps. This means that no artificial boundaries between adjacent 50-trial windows were introduced, ensuring a continuous representation of state fluctuations. The trials were then concatenated across days per mouse (e.g., 500 trials × 14 d = 7,000 concatenated trials), with no additional smoothing applied, ensuring that smoothing remained independent between training days. The resulting data were finally partitioned into trial count histograms with 100 bins (Fig. 2*B*, top). Trials at or below the second bin of each animal's licking rate were classified as “low/no licking.” All other trials were classified as “moderate/high licking” (Fig. 2*B*, top).

*Correctness*. A correctness percentage vector (length, 500 trials) was constructed by smoothing binary go/no-go correctness data (hits or correct rejections, “1”) using a 50-trial moving mean progressing in 1-trial steps independently within each training day (Fig. 2*A*, bottom), following the same rationale for state variations as described above. These vectors were concatenated across days per mouse (e.g., 500 trials × 14 d = 7,000 concatenated trials) and partitioned into trial count histograms with 100 bins (Fig. 2*B*, bottom). Trials at or above the 75th quantile of each animal's correctness were classified as “good performance”, to accommodate inter-animal differences in correctness distributions, as detailed below. All other trials were classified as “chance performance” (Fig. 2*B*, bottom).

*Tripartite state categorization*. The “persistent” category consisted of trials with both “moderate/high licking activity” and “chance performance.” The choice to use “persistent” to denote this state is because it is an objective term, agnostic to the underlying process: whether exploration, impulsivity, or a combination of both, in alignment with other studies (Parent et al., 2015; Amarante et al., 2017). In turn, the “disengaged” category consisted of all trials with “low/no licking activity.” Finally, the “attentive” category consisted of “good performance” trials (Fig. 2*C*). These categories were defined without any form of trained classification and are here termed “original,” thus distinguishing from the “predicted” categories explained in the next subsection.

Supplementarily, Extended Data Figure 2-1 shows lick rate histograms from all mice. As illustrated in these histograms, the lowest lick rate bin was always dominant and could have been considered sufficient to characterize “disengaged” states. However, the choice here was to also include the second lowest lick rate bin in “disengaged” states, in order to account for periods of infrequent licking that sometimes occurred within otherwise “disengaged” states (Extended Data Fig. 1-2). These brief bursts of activity occurred sporadically during task disengagement, often accompanied by wheel running with no consistent timing relative to go/no-go cueing (Extended Data Fig. 1-2). These task-unrelated wheel running behaviors were not analyzed separately, as they were infrequent and peripheral to the scope of this study.

Also supplementarily, Extended Data Figure 2-1 shows correctness percentage histograms from all mice. According to these histograms, all mice included here learned the task across days of training, but the timing and incidence of peak correctness varied both among mice and within sessions. The distribution of correctness values (one value per trial) also differed among mice, including variations in range, skew, and bimodality (Extended Data Fig. 2-1). Thus, a distribution parameter appropriate for one mouse (e.g., an antimode between poor and good task performance) was not necessarily optimal for another, hence the use of a 75th quantile correctness cutoff per mouse, as explained above. With this approach, within-subject learning was emphasized; applying a fixed correctness cutoff for all mice would risk underrepresenting “attentive” states in lower-performing mice.

#### State prediction based on nonperformance variables

This step involved individualized (within-mouse) decision-tree classifiers trained on eight nonperformance variables, i.e., motor and physiologic activity variables outside whole-trial lick rates and go/no-go correctness (Fig. 2*D*). All nonperformance variables consisted of vectors produced with similar methods as described above for Figure 2*A*–*C*, including 500 trials per daily session, 50-trial moving averages with 1-trial steps to capture performance fluctuations on the scale of minutes to tens of minutes, and trial concatenation across days (e.g., 500 trials × 14 d = 7,000 concatenated trials). See also Extended Data Figure 1-2 for examples of fluctuations in nonperformance variables.

*Prestimulus lick rate*. The number of licks during the 1 s prestimulus period, capturing state-driven licking before go/no-go decisions or consummatory licking.

*Whole-trial wheel speed, pupil diameter, and eyelid aperture*. Averages across the 5 s trial period, summarizing peristimulus locomotor reactions and eye activity.

*Prestimulus PSS*. Mean PSS across probe channels during the 1 s prestimulus period, capturing brain state devoid of electromyographic noise from consummatory licking.

*Stimulus-evoked LFP response*. Mean LFP amplitude across probe channels during the 200 ms stimulus, capturing electrophysiological reactions immediately prior to licking responses.

*Prestimulus MUA power*. Same as prestimulus PSS but from mean MUA power.

*Stimulus-evoked MUA response*. Same as prestimulus PSS but from mean MUA.

The classifiers were trained individually per mouse, based on input tables where columns represented the nonperformance variables listed above, and rows represented concatenated trials from all days. For example, a mouse with 14 daily sessions would result in a table with eight columns (nonperformance variables) and 7,000 rows (14 sessions × 500 trials). Accompanying the table was a categorical vector with the original labels of “persistent,” “disengaged,” or “attentive,” one label per trial. To deconstruct 50-trial moving average patterns (see smoothing methods above), the rows (not the columns) of the table were shuffled in blocks of 25 trials, which additionally helped randomize the chronological order of trials. The shuffled table was then split into training and heldout sets (1:1) and vice versa in a separate run (Fig. 2*E*, top). The predictions from the two heldout sets were finally recombined into chronological order, forming a matrix (14 sessions × 500 trials) of predicted “persistent,” “disengaged,” and “attentive” trials (Fig. 2*E*, bottom).

Classification consisted of a bagged decision-tree method via the *fitcensemble* function with the following parameters: method, “Bag”; number of learning cycles, 30; and learner, a template tree with maximum number of decision splits set to *n* − 1 (where *n* is the number of trials). These functions and parameters were determined through exploratory analysis using the Classification Learner application in MATLAB (MathWorks) and then incorporated into custom code.

#### ROC AUC-based evaluation

To evaluate model accuracy, receiver operating characteristic (ROC) area under the curve (AUC) values were obtained using the *perfcurve* function. Specifically, the classification method above was iterated in groups of 100 iterations, each group using a different pool (1–8) of randomly selected nonperformance variables (Fig. 2*F*–*H*) or a single nonperformance variable, for context (Fig. 2*I,J*). ROC false- and true-positive rates were aggregated per group of 100 iterations and plotted along with their corresponding AUC for qualitative illustration (Fig. 2*F*,*G*,*I*). For quantification of such AUC values, they were plotted as a function of the groups of iterations—from less (1) to more (8) diverse pool of variables—resulting in asymptotic curves (Fig. 2*H*). Quantifying these curves required nonlinear least-squares fitting to an asymptotic exponential model, ultimately providing a coefficient of determination (*R*^2^) and a *p* value for the asymptotic rate parameter *k*. Thus, the lower the *p* value, the greater the confidence in the asymptotic trend (Fig. 2*H*). The same was then performed on single-variable iterations, again for context (Fig. 2*J*). See also Extended Data Figure 2-2 for ROC AUC analyses per mouse.

#### Supplemental controls for state analysis

To support the individualized state analysis above, supplemental prediction iterations were tested, interrogating whether one mouse's decision tree could predict another mouse's states (Extended Data Fig. 2-3). The methods were identical to those previously outlined, except for one difference in the dataset splitting step (Fig. 2*E*). When splitting the shuffled input table into training and heldout sets (1:1), a heldout set of the same size (i.e., same number of trials, same variables) was taken from a different mouse and vice versa in a separate run. Predictions from each heldout set were then reassigned to their original mice in a chronological order of trials. All possible predictor-predicted mouse pairs were tested, for comprehensiveness. Quantifications comparing between-mouse predictions and within-mouse predictions were then made using ROC AUC, resulting in supplemental data (Extended Data Fig. 2-3). For additional context for state analysis, regression methods were used and reported here as alternatives to decision-tree classification (Extended Data Fig. 2–4). One such method consisted of generalized linear models (GLM; *fitglm* function) conducted in leave-one-predictor-out iterations: for each GLM iteration, one predictor (i.e., nonperformance) variable was omitted, and *t* statistic values were cumulatively obtained for all other predictors, resulting in seven *t* statistic values per predictor (i.e., total number of predictors minus the omitted one). These values were then cumulatively plotted across mice, forming “violin” distributions (Extended Data Fig. 2-4). Another method consisted of multinomial regressions (MNR; *fitmnr* function), also conducted in leave-one-predictor-out iterations (Extended Data Fig. 2-4). The main difference between these two approaches resided in the predicted (or response) variable: task correctness for GLM and state categories for MNR. The rationale is that GLM is appropriate for predicting a continuous outcome such as task correctness, whereas MNR is suited for classifying categories, like the states studied here. Both approaches (GLM and MNR) were each conducted again but using shuffled response variables, enabling real-data versus shuffled-data comparisons, statistically quantified using Friedman's test (Extended Data Fig. 2-4).

Finally, other controls for state analysis are shown in a supplement using the same decision-tree and ROC AUC methods above but exploring alternative approaches (Extended Data Fig. 2-5). First, the specific contribution of prestimulus licking to state prediction was assessed using decision-tree iterations that either consistently included or consistently excluded this variable (Extended Data Fig. 2-5). Second, two subsets of predictor variables were separately examined for their influence on state prediction: physiological/behavioral variables (wheel, licking, and eye activity) and neurophysiological variables (LFP- and MUA-derived metrics; Extended Data Fig. 2-5). Third, data shuffling procedures were compared, contrasting temporal shuffling (random permutation of trial sequence, i.e., just the rows of the data tables) with full shuffling (random permutation of both trial sequence and predictor variables, i.e., both rows and columns; Extended Data Fig. 2-5). Fourth, different trial smoothing window sizes (1, 10, and 50 trials) were tested, illustrating that 50-trial smoothing was better able to capture the timescale of interest to this study than shorter windows (Extended Data Fig. 2-5).

#### Motoric signs of learning within states and trial outcomes

**Figure 3.**
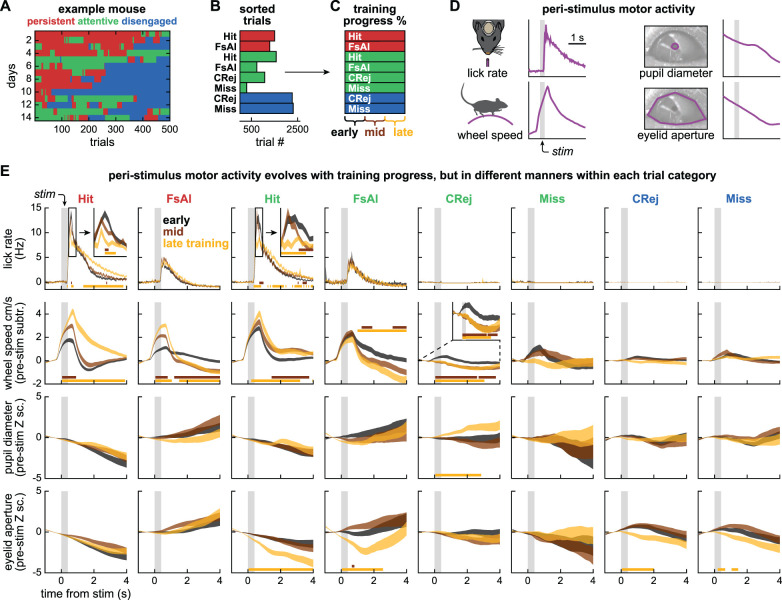
Learning-like changes in motor activity within each state. ***A***, Day-by-trial matrix of state categories from another representative mouse (similar to an example shown previously in Fig. 2*C*). ***B***, For the analysis in this figure, state categories were further sorted by go/no-go outcome, resulting in eight subcategories of trials. The chronological order of trials was preserved within each subcategory. Infrequent trial combinations (i.e., hits and false alarms during “disengaged” state, correct rejections, and misses during “persistent” state) were excluded. ***C***, Trials were divided into a common number of blocks (99) per subcategory, creating a “normalized” training progression axis. These trial blocks were then grouped into early, mid, and late-training stages (33 blocks each) and aggregated across mice for quantification (*n* = 5). ***D***, Schematics showing the four types of peristimulus motor activity analyzed across training stages per state subcategory. ***E***, Changes in peristimulus patterns from early to late training (curve colors) were examined for motor activity variables (rows of graphs) and trial subcategories (columns of graphs). The data are shown as ±standard error curves, including post hoc comparisons between learning stages along the peristimulus time axes (dark brown, early vs mid; light brown, early vs late), following two-way ANOVA with repeated measures (Table 1). These data reveal signs of learning outside whole-trial lick rate and correctness metrics.

Trials that had been sorted into state categories (Fig. 3*A*) were further sorted by go/no-go outcome, maintaining the chronological order within each subcategory (Fig. 3*B*). This trial sorting method was applied individually to each mouse. This resulted in uneven distributions of go/no-go outcomes across state states (Fig. 3*B*): trials labeled as “persistent” mostly ended in attended trials, i.e., hits or false alarms (74%), whereas trials labeled as “disengaged” mostly ended in unattended trials, i.e., correct rejections or misses (97%), as expected. Only the “attentive” state showed relatively balanced proportions of attended (58%) and unattended (42%) trials. Hence, unattended trials during “persistent” and attended trials during “disengaged” states were excluded from this analysis, resulting in eight subcategories of trials (Fig. 3*B*). Each subcategory was then divided into 99 blocks of trials, resulting in a common (“normalized”) training progression scale per mouse (Fig. 3*C*). Each training progression block was then examined for peristimulus patterns in lick rate, wheel speed, pupil diameter, and eyelid aperture (1 s prestimulus, 4 s poststimulus; Fig. 3*D*).

Peristimulus patterns were averaged per block of trials, along the 99-block training progression scale explained above (Fig. 3*C*). These blocks were divided into three equal stages: early (initial 33 blocks), mid (middle 33 blocks), and late training (last 33 blocks), leading to the color-coded curves in Figure 3*E*, with shaded curves depicting ±standard error across mice and trial blocks. Statistical differences between training stages were quantified using two-way ANOVA with peristimulus time samples as repeated measures (Table 1) and illustrated by post hoc differences in the subplots, using Tukey's protection for pairwise comparisons (Fig. 3*E*). To emphasize stimulus-evoked patterns over basal ongoing activity, wheel activity was mean-subtracted, and eye activity was *Z*-scored relative to their prestimulus periods. Specifically for wheel speed, mean subtraction was used instead of *Z*-scoring, as *Z*-scoring was observed to exaggerate the small activity peaks in correct rejections and misses (Fig. 3*E*). For eye activity, *Z*-scoring was necessary to reveal subtle slow trends (Fig. 3*E*).

**Table 1.** Statistics for Figure 3*E*

			Impulsive		Attentive				Disengaged	
Variable	Effect	Value	Hit	Fs alarm	Hit	Fs alarm	Corr rej	Miss	Corr rej	Miss
Lick rate	Training stage	Deg Fr	2,328	2,328	2,328	2,328	2,328	2,328	2,328	2,328
		*F*	25.874	5.305	7.444	0.329	0.547	0.716	0.662	1.433
		*P*	**<0.001**	**0.005**	**<0.001**	0.72	0.579	0.489	0.517	0.24
	Peristim time	Deg Fr	79,12956	79,12956	79,12956	79,12956	79,12956	79,12956	79,12956	79,12956
		*F*	377.608	274.844	227.873	70.687	4.063	1.522	1.143	1.889
		*P*	<0.001	<0.001	<0.001	<0.001	<0.001	0.002	0.181	<0.001
	Interaction	Deg Fr	158,25912	158,25912	158,25912	158,25912	158,25912	158,25912	158,25912	158,25912
		*F*	11.57	3.634	9.308	1.609	1.236	1.101	1.06	1.609
		*P*	**<0.001**	**<0.001**	**<0.001**	**<0.001**	0.024	0.184	0.288	**<0.001**
Wheel speed	Training stage	Deg Fr	2,328	2,328	2,328	2,328	2,328	2,328	2,328	2,328
		*F*	54.805	3.654	9.635	7.85	7.375	1.113	3.196	0.16
		*P*	**<0.001**	0.027	**<0.001**	**<0.001**	**<0.001**	0.33	0.042	0.852
	Peristim time	Deg Fr	79,12956	79,12956	79,12956	79,12956	79,12956	79,12956	79,12956	79,12956
		*F*	171.024	236.512	132.777	119.43	21.249	10.752	2.289	13.97
		*P*	<0.001	<0.001	<0.001	<0.001	<0.001	<0.001	<0.001	<0.001
	Interaction	Deg Fr	158,25912	158,25912	158,25912	158,25912	158,25912	158,25912	158,25912	158,25912
		*F*	29.043	44.345	4.046	4.048	0.653	4.154	1.794	6.779
		*P*	**<0.001**	**<0.001**	**<0.001**	**<0.001**	1	**<0.001**	**<0.001**	**<0.001**
Pupil diameter	Training stage	Deg Fr	2,328	2,328	2,328	2,328	2,328	2,328	2,328	2,328
		*F*	1.529	2.635	0.383	0.897	5.884	0.397	0.557	1.532
		*P*	0.218	0.073	0.682	0.409	**0.003**	0.673	0.573	0.218
	Peristim time	Deg Fr	79,12956	79,12956	79,12956	79,12956	79,12956	79,12956	79,12956	79,12956
		*F*	59.459	18.839	22.365	6.495	0.268	2.518	6.927	5.02
		*P*	<0.001	<0.001	<0.001	<0.001	1	<0.001	<0.001	<0.001
	Interaction	Deg Fr	158,25912	158,25912	158,25912	158,25912	158,25912	158,25912	158,25912	158,25912
		*F*	2.954	2.007	1.496	0.263	3.625	1.167	2.065	1.264
		*P*	**<0.001**	**<0.001**	**<0.001**	1	**<0.001**	0.075	**<0.001**	0.014
Eyelid aperture	Training stage	Deg Fr	2,328	2,328	2,328	2,328	2,328	2,328	2,328	2,328
		*F*	2.895	1.174	12.266	6.81	1.075	0.885	6.524	6.675
		*P*	0.057	0.31	**<0.001**	**0.001**	0.343	0.414	**0.002**	**0.001**
	Peristim time	Deg Fr	79,12956	79,12956	79,12956	79,12956	79,12956	79,12956	79,12956	79,12956
		*F*	83.092	35.613	44.226	8.931	3.063	3.375	19.964	32.366
		*P*	<0.001	<0.001	<0.001	<0.001	<0.001	<0.001	<0.001	<0.001
	Interaction	Deg Fr	158,25912	158,25912	158,25912	158,25912	158,25912	158,25912	158,25912	158,25912
		*F*	1.522	1.302	3.234	1.387	1.113	1.091	1.37	1.269
		*P*	**<0.001**	**0.007**	**<0.001**	**<0.001**	0.158	0.206	**0.001**	0.013

Rows of variables (lick rate, wheel speed, pupil diameter, eyelid aperture) and columns (arousal states and go/no-go outcomes) are organized as in the figure. Degrees of freedom, *F*, and *p* values were obtained using two-way ANOVA with peristimulus time bins as repeated measures. *p* values lower than 0.01 are highlighted in bold font, except in the effects of peristimulus time.

#### Electrophysiologic signs of learning within states and trial outcomes

**Figure 4.**
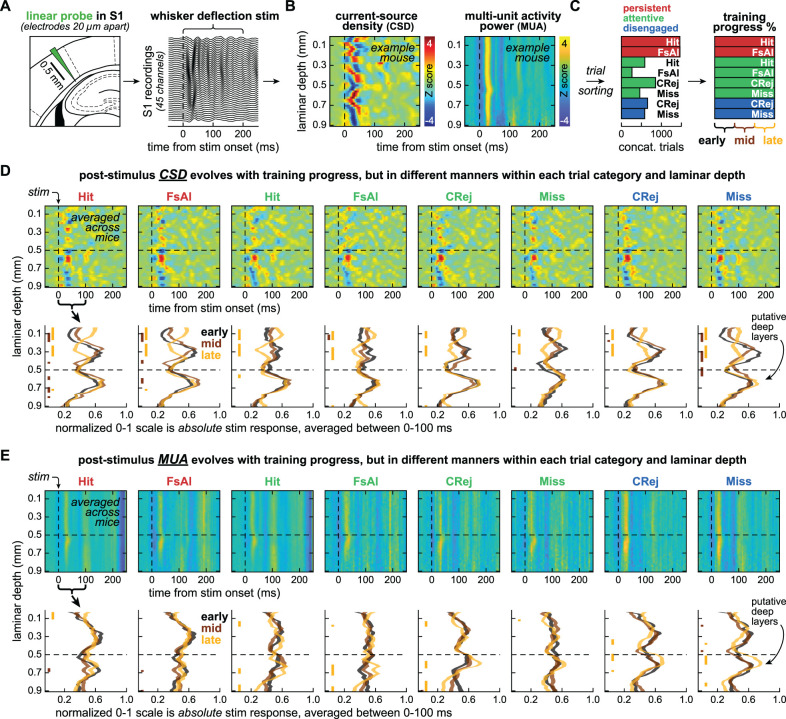
Learning-related changes in neocortical laminar activity within each state. ***A***, A 64-channel linear probe (20 µm between recording sites) was implanted into the S1 cortex. The top 45 channels—0.9 mm, roughly aligning with the S1 cortex—were analyzed in 300 ms peristimulus epochs. ***B***, Each epoch was processed into a pair of channel-by-time matrices: CSD and MUA power, *Z*-scored horizontally. ***C***, Using the same methods as Figure 3, *B* and *C*, CSD and MUA matrices of each trial were sorted into state and go/no-go outcome subcategories and averaged into 99 trial blocks within each subcategory. These trial blocks were then grouped into early, mid, and late training (33 blocks each) and aggregated across mice for quantification (*n* = 3 in this analysis). ***D***, The heatmaps (top) show *Z*-scored CSD, averaged per trial subcategory across mice (same color bar as panel ***B***). The curves (bottom) show neocortical laminar profiles, processed by converting CSD data to absolute values, averaging these values per channel across 0–100 ms poststimulus time, and normalizing the results to a common 0–1 scale. The data are shown as ±standard error curves, including post hoc comparisons between learning stages along the peristimulus time axes (dark brown, early vs mid; light brown, early vs late), following two-way ANOVA with repeated measures (Table 2). ***E***, Same as panel ***D*** but for MUA power. Overall, plasticity-like changes were observed from early to late training, with certain features of the neocortical laminar profiles changing more prominently depending on the trial subcategory. See also Extended Data Figure 4-1.

Stimulus-evoked electrophysiological responses were extracted per trial (50 ms before, 250 ms after stimulus onset), producing channel × time matrices (Fig. 4*A*). Here, the top 45 channels of the silicon probes were examined, spanning ∼0.9 mm in the S1 cortex (Fig. 4*A*). Either of two sampling rates was used to extract these epochs: 1,250 Hz for CSD and 20 kHz for MUA power (Fig. 4*B*). CSD was obtained by calculating the second derivative across channels, followed by *Z*-scoring per channel, spatial smoothing (five-channel window), and temporal smoothing (20 ms window; Fig. 4*B*, left). For MUA power, one spectrogram was generated per channel (*spectrogram* function) using the following parameters: frequency range, 500–5,000 Hz; 500 Hz bin size (linearly spaced); and window, 4 ms (50% overlap). The sum of absolute MUA power across frequency bins was then calculated, resulting in one peristimulus MUA power curve per channel. The resulting channel × time MUA power matrices were *Z*-scored per channel and temporally smoothed (10 ms window), without spatial smoothing (Fig. 4*B*, right). CSD and MUA power matrices were finally sorted by trial subcategory (i.e., the combination of state and go/no-go outcome) and training progression (Fig. 4*C*), as in the methods described earlier for peristimulus motor activity (Fig. 3*B*,*D*).

For quantification (Fig. 4*D*,*E*), each CSD and MUA power matrix was converted to absolute magnitudes, so that negative and positive values (e.g., sinks and sources) contributed equally to measure the strength of stimulus responses. These absolute values were then averaged across time samples, particularly the 0–100 ms period after stimulus onset, thus emphasizing the maximal CSD and MUA responses (see heatmaps in Fig. 4*D*,*E*, averaged per trial subcategory across mice and training phases). This resulted in laminar depth profiles, which were mean-subtracted (22-channel window) to remove large trends and then normalized to a 0–1 axis. With these processing steps, laminar features on the scale of ∼0.2 mm were revealed (Fig. 4*D*,*E*, curves). Such features were analyzed in terms of training progress, motivation states, and trial outcomes, like the previous analysis of peristimulus motor activity (Fig. 3*E*). The shaded curves in Figure 4, *D* and *E*, depict ±standard error across trial blocks and mice (*n* = 3 in this analysis). Statistical differences between training stages were quantified using two-way ANOVA with laminar depths as repeated measures (Table 2) and illustrated by post hoc differences in the subplots, using Tukey's protection for pairwise comparisons (Fig. 4*D*,*E*).

**Table 2.** Statistics for Figure 4*D,E*

			Impulsive		Attentive				Disengaged	
Variable	Effect	Value	Hit	Fs alarm	Hit	Fs alarm	Corr rej	Miss	Corr rej	Miss
Poststim laminar CSD strength	Training stage	Deg Fr	2,196	2,196	2,196	2,196	2,196	2,196	2,196	2,196
		*F*	6.938	1.216	1.664	0.156	1.595	0.099	1.939	2.150
		*P*	**0.001**	0.299	0.192	0.855	0.206	0.906	0.147	0.119
	Depth (channels)	Deg Fr	44,4312	44,4312	44,4312	44,4312	44,4312	44,4312	44,4312	44,4312
		*F*	58.092	67.273	44.323	24.023	79.295	26.054	81.700	65.167
		*P*	<0.001	<0.001	<0.001	<0.001	<0.001	<0.001	<0.001	<0.001
	Interaction	Deg Fr	88,8624	88,8624	88,8624	88,8624	88,8624	88,8624	88,8624	88,8624
		*F*	18.137	17.122	8.507	6.237	7.646	7.216	8.851	12.358
		*P*	**<0.001**	**<0.001**	**<0.001**	**<0.001**	**<0.001**	**<0.001**	**<0.001**	**<0.001**
Poststim laminar MUA strength	Training stage	Deg Fr	2,196	2,196	2,196	2,196	2,196	2,196	2,196	2,196
		*F*	1.441	2.659	0.852	0.643	1.290	2.838	1.500	2.411
		*P*	0.239	0.073	0.428	0.527	0.278	0.061	0.226	0.092
	Depth (channels)	Deg Fr	44,4312	44,4312	44,4312	44,4312	44,4312	44,4312	44,4312	44,4312
		*F*	28.002	21.396	19.343	5.267	31.430	10.079	42.980	44.851
		*P*	<0.001	<0.001	<0.001	<0.001	<0.001	<0.001	<0.001	<0.001
	Interaction	Deg Fr	88,8624	88,8624	88,8624	88,8624	88,8624	88,8624	88,8624	88,8624
		*F*	4.366	2.449	5.516	5.409	6.014	3.195	6.579	7.916
		*P*	**<0.001**	**<0.001**	**<0.001**	**<0.001**	**<0.001**	**<0.001**	**<0.001**	**<0.001**

CSD, current-source density; MUA, multiunit activity. Rows of variables (CSD and MUA) and columns (arousal states and go/no-go outcomes) are organized as in the figure. Degrees of freedom, *F*, and *p* values were obtained using two-way ANOVA with laminar depths as repeated measures. *p* values lower than 0.01 are highlighted in bold font, except in the effects of depth.

Supplementarily, trial subcategories were compared within each training stage (Extended Data Fig. 4-1). Laminar depth channels encompassing the strongest training-related changes—superficial CSD (0.20–0.36 mm) and deep MUA (0.50–0.64 mm), according to the main data (see Results section; Fig. 4*D*,*E*)—were averaged per trial block, thus eliminating the laminar depth dimension. This facilitated direct comparisons among trial types, displayed as violin distributions (Extended Data Fig. 4-1) and quantified statistically using Friedman's test, followed by post hoc comparisons with Tukey's protection.

A laminar depth feature in MUA, in particular, was relevant for data exclusion. Specifically, to verify the alignment of laminar depth profiles across mice, a feature in laminar MUA was used as spatial reference, characterized by increased MUA ∼0.6 mm depth (Fig. 4*E*), similar to electrophysiological landmarks previously reported in the mouse visual cortex (Senzai et al., 2019). Two of the five mice used here did not exhibit this laminar feature and were therefore excluded from this laminar profile analysis. These two mice still contributed nonlaminar, channel-averaged electrophysiological data for state analysis (e.g., prestimulus PSS and stimulus-evoked LFP responses, explained above; Fig. 2) and behavioral/physiological data (Figs. 1–3). This limitation is also acknowledged in the Discussion section.

### Histology

After experiments, mice were deeply anesthetized with isoflurane, and electrolytic lesion currents (50 µA, 10 s, against ground) were applied through the silicon probe at every eighth channel. Mice were then subjected to standard transcardiac perfusion techniques, including blood clearing of the brain with phosphate-buffered saline (PBS) followed by fixation of the brain with 4% paraformaldehyde (PFA) in PBS. Brains were extracted from the skull and stored in 4% PFA. One day before sectioning, brains were transferred to PBS. Coronal sections (60 µm thick) were cut using a vibratome and mounted onto gelatin-coated slides. Specimens were air-dried for 12–24 h and processed via standard Nissl staining, including tissue defatting (xylenes), rehydration (ethanol 100–50%), Cresyl violet staining, and dehydration (ethanol 50–100%). Finally, specimens were coverslipped using mounting medium and imaged using a bright-field microscope (Motic). See Extended Data Figure 1-1.

### Code accessibility

The code for classification of motivation states was developed interactively using the Classification Learner App in MATLAB and is freely available at https://github.com/brendonw1/WatsonLabCode/tree/master/LearningMotivationStudy.

## Results

### Experimental design, summary of dataset, and study objects

As elaborated in Materials and Methods, head-fixed mice were trained to discriminate whisker deflection stimuli (Schriver et al., 2018) while being monitored for licking, wheel, eye, and brain activity (Fig. 1*A*,*B*). The training consisted of 12–14 daily sessions with pseudorandom sequences of 500 go/no-go trials (Fig. 1*C*). Each trial included fixed peristimulus periods (prestimulus, stimulus, answer, and intertrial) and resulted in standard go/no-go outcomes: hit (rewarded with water), miss, correct rejection, and false alarm (punished with trial extension; Fig. 1*D*). Brain activity was recorded using a linear silicon probe across the S1 cortex, after imaging-guided implantation (Extended Data Fig. 1-1) and whisker-barrel somatotopy check.

10.1523/ENEURO.0417-25.2026.f1-1 Figure 1-1 **Intrinsic optical imaging maps for further specification of S1 cortex implantation coordinates. A,** Trial-averaged images from a representative mouse showing surface hemodynamics during and after piezoelectric stimulation of a whisker pair. A thinned-skull window (see coordinates in millimeters) was imaged under high-power red light (630 nm) at 4 Hz for 12 s: 4 s before (baseline, not shown), 4 s during and 4 s after stimulation. Parenchymal and blood vessel signals were magnified by normalizing during- and post-signal images against the baseline mean (see percentage colorbar), followed by trial averaging (40 trials per whisker pair). Methods were based on previous studies (Aronoff and Petersen, 2007). **B,** For each mouse, a single blood vessel image was obtained under high-power green light (525 nm) and overlaid on the grand-average S1 barrel signal, creating a map to guide future silicon probe implantation. The signal in orange indicates >2 standard deviations from the mean. **C,** Examples of S1 barrel imaging from strong to dim, along with horizontally averaged cortical laminar responses (current-source density, CSD) to whisker stimulation in the awake mouse (see main manuscript, Fig. 4), as well as Nissl-stained coronal sections showing electrolytic lesion along the silicon probe tract. Download Figure 1-1, TIF file.

10.1523/ENEURO.0417-25.2026.f1-2 Figure 1-2 **Qualitative illustrations from a single mouse, showing variations in behavioral and physiological metrics during two representative sessions: early and late training.** All curves represent whole-trial measures (one value per trial) in chronological order (x axes), analyzed with a 50-trial moving mean. Specifically in licking and wheel activity (second and third rows of graphs), whole-trial measures (right-side y axes, blue curves) are overlaid on raster plots and heatmaps displaying peri-stimulus patterns (left-side y axes). Both longer-term trends and smaller fluctuations can be observed across graphs, with varying levels of redundancy among variables. These inter-variable relationships were examined using linear classification in the main manuscript (Fig. 2). Download Figure 1-2, TIF file.

Figure 1*E* shows daily averages from all mice. Learning is evidenced by an upward trend in correctness. Other ascending curves were observed in wheel speed, PSS from LFP, and MUA power (Fig. 1*E*), suggesting increase in motor activity and high-frequency brain activity across days. Licking and eye activity showed no significant slope over the days and/or varied across animals, indicating nonredundancy in the metrics. Body weight also showed no clear trend, suggesting that appetitive motivation did not uniformly affect the metrics (Fig. 1*E*).

Figure 1*F* exemplifies correctness and lick rate variations within and across training sessions. In this study, these variations were assessed as indicators of a “learning-motivation continuum,” with each mouse showing a unique continuum. These variations are also qualitatively illustrated in Extended Data Figure 1-2, along with variations in wheel running, pupil diameter, eyelid aperture, and neocortical PSS and MUA power.

### Performance and nonperformance metrics covary with learning and motivation state

Figure 2*A* displays two day-by-trial matrices, each matrix with 14 training sessions (rows) and 500 trials (columns), both from the same animal. One matrix shows whole-trial lick rates, and the other shows correctness percentages, both with a 50-trial moving mean (one-trial steps). Within each day, high lick rates generally preceded periods of low/no licking, with these state changes typically occurring around Trials 250–350 (Fig. 2*A*, top). In turn, periods of elevated correctness generally emerged around Trials 200–300, after 4–6 d of training (Fig. 2*A*, bottom). However, additional variation was observed. For example, on Day 8, this mouse exhibited sustained high correctness from the session start until around Trial 400, whereas on Day 14, the same mouse maintained high lick rates throughout the session but with inconsistent correctness (Fig. 2*A*). These variations occurred despite regular laboratory routine, suggesting that state variations were at least in part spontaneous, including in well-trained animals (Yin et al., 2025).

10.1523/ENEURO.0417-25.2026.f2-1 Figure 2-1 Licking and task correctness curves and histograms from all mice (supplementing Figs. 1*F* and 2A,*B*). A, Trials were concatenated across days (500 trials/day), illustrating variations within and between sessions. Each session was independently smoothed using a 50-trial moving mean. Color codes indicate distinct trial categories for lick rates and correctness, which were later combined into the tripartite state category system used in the main analysis (Fig. 2*C*). **B,** The same data and color coding are shown as trial count histograms, each with a fixed number of bins (100) and variable bin edges. See Methods for rationale regarding lick rate and correctness cutoffs. Download Figure 2-1, TIF file.

10.1523/ENEURO.0417-25.2026.f2-2 Figure 2-2 **Supplementary individual-mouse illustrations reinforcing that performance and non-performance metrics covary with learning and arousal. A,** Same receiver-operating characteristic (ROC) analysis as in the main manuscript (Fig. 2*F*) but depicting all mice in the rows of graphs. Linear classification of arousal states was conducted in eight groups of 100 iterations, each group using a different number of randomly-chosen non-performance predictors. Cumulative true positive and false positive rates per state were then calculated per group of 100 iterations, showing that the more diverse the pool of non-performance predictors, the better the state classification. **B,** Area under the curve (AUC) analysis from the same ROC data, showing asymptotic prediction improvement with increasing numbers of non-performance predictors. Prediction improvement was generally more evident for the impulsive and attentive state categories (see R-squared correlations). **C,** Day-by-trial state category matrices per mouse, for qualitative comparison between original and predicted categories. In this case, predictions were obtained from all eight non-performance variables. **D-F,** Similar layout, but using single-variable iterations, ranked by mean AUC across states. No single variable matched the classification performance obtained using multiple variables. Download Figure 2-2, TIF file.

10.1523/ENEURO.0417-25.2026.f2-3 Figure 2-3 **State predictions within each individual mouse and across all possible mouse pairs. A,** Grid of receiver operating characteristic (ROC) subplots. All ROC curves were generated using the full set of eight non-performance variables. The diagonal shows within-mouse analysis, where both training and held-out data are from the same mouse. Off-diagonal subplots represent “predictor-predicted” mouse pairs, with training data from one mouse and held-out data from another (see Methods). **B,** AUC values from the ROC subplots in panel A, showing consistently higher predictive accuracy for individualized (within-mouse) state predictions compared to cross-mouse predictions, especially for attentive states. Download Figure 2-3, TIF file.

10.1523/ENEURO.0417-25.2026.f2-4 Figure 2-4 **Alternative analysis of motivation states using Generalized Linear Models (GLM) and Multinomial Regression (MNR). A,** Violin distributions of GLM t-statistic values (filled datapoints) and their medians (white datapoints) per predictor variable (y-axis). These t-statistic values were cumulatively plotted across mice, each mouse represented by multiple leave-one-predictor-out GLM iterations, where one predictor variable is omitted per iteration (intercept always removed). The response variable used in this analysis was task correctness percentage (see Figs. 1*F* and 2*A*,*B*, as well as Extended Data Fig. 2-1), thus testing the ability of these GLMs to predict task correctness independent of state categories. The same leave-one-predictor-out iterations were conducted for shuffled correctness percentage data, generating controls (black datapoints) for statistical comparisons using Friedman’s test (see chi-square and P values). Asterisks indicate post-hoc differences (P < 0.005) between real-data and shuffled-data iterations, using Tukey’s protection for pairwise comparisons. **B,** Similar analysis as panel A, but using leave-one-predictor-out MNR iterations for predicting the three state categories of the main study. In MNR, one category is always used as reference for the other categories. Thus, an additional iteration level was necessary for MNR, so that each of the three states was assigned as the reference category (i.e., one reference state per turn). T-statistic values resulting from these two MNR iteration levels (leave-one-predictor-out and reference categories) were parsed by motivation state, as shown in these three plots. Insets additionally show the medians of violin distributions, magnifying subtle differences between real-data and shuffled-data iterations. These MNR results help “decompose” the GLM patterns observed in panel A. For instance, negative t-statistic values for pre-stimulus licking and positive values for pre-stimulus MUA power in panel A can be attributed to disengaged and attentive states, respectively, given the results from MNR. Thus, these GLM and MNR analyses complement each other while reinforcing the scope of the main study, focused on state categories. **C,** Same medians and post-hoc differences as panel B, but plotted on a shared t-statistic axis for qualitative comparison among states. Download Figure 2-4, TIF file.

10.1523/ENEURO.0417-25.2026.f2-5 Figure 2-5 **Several additional controls for the receiver operating characteristic (ROC) area under the curve (AUC) analysis in Fig. 2. A,** Pre-stimulus licking was identified as the most influential predictor variable in Fig. 2*I*,*J*. Here, its impact on predictive accuracy was further tested through ROC-AUC iterations that either always omitted or always included this variable. When pre-stimulus licking was always omitted, the AUC curve asymptotes remained strong, but their plateaus decreased slightly, reaching 0.86 AUC for the persistent/attentive states compared to 0.89 AUC in the main analysis (Fig. 2*H*). Conversely, when pre-stimulus licking was always included, the asymptotic trends became weaker, and AUC values plateaued at the same level as the main analysis (Fig. 2*H*). Importantly, the attentive state remained the most challenging to predict in this supplementary analysis (see also Fig. 2*I*,*J*). These observations clarify that pre-stimulus licking showed partial reliability at predicting persistent and disengaged states, and insufficient reliability at predicting attentive states. **B,** Same ROC AUC analysis, but using two subsets of predictors: physiological/behavioral (pre-stimulus lick rates, and whole-trial wheel speed, pupil diameter and eyelid aperture) and neurophysiological (pre-stimulus PSS / MUA power, and channel-averaged stimulus-evoked LFP / MUA responses). Results revealed a modest difference between subsets; for the attentive state, the most difficult to predict, the physiology/behavior subset plateaued at 0.84 AUC versus 0.82 AUC for the neurophysiology subset. Asymptotic trends showed P values < 0.03 in all cases, though these values are interpreted with caution due to the limited number of datapoints per asymptote (four compared to eight in the main analysis in Fig. 2). This reinforces that combinations of four or more variables – whether behavioral, physiological, or neurophysiological – can better predict learning and intra-task states in this and potentially other similar experiments. **C,** In the main decision-tree analysis (Fig. 2), the predictor tables (trials in rows, predictors in columns) were shuffled only across rows, deconstructing the chronological order of trials. Here, comparisons were made between these row-shuffled data and fully shuffled data, where both trial sequences and inter-variable relationships were randomized. Individual AUC values from these experimental conditions (one value per decision-tree iteration) were plotted as violin distributions and statistically compared using Friedman’s test (see chi-square and P values). Supporting the main study, trial shuffling led to asymptotic AUC plateauing as the number of predictors increased, whereas full shuffling consistently yielded AUC around 0.5. **D,** Similar ROC AUC analysis, now focused on the full predictor set (all eight non-performance variables) to assess shorter trial smoothing windows. As explained in the Methods, whole-trial lick rates and task correctness were smoothed using 50-trial moving windows revealing motivation state fluctuations, similar to a previous study (Matteucci et al., 2022) and unlike other studies more interested in rapid processes over fewer trials, like arousal states and decision making (Ashwood et al., 2022; Hulsey et al., 2024). This panel supplements this rationale by presenting ROC AUC results from unsmoothed and 10-trial smoothed licking and correctness data. Download Figure 2-5, TIF file.

Here, these variations were sorted into motivation states (Fig. 2*B*,*C*), instead of assigning these states to fixed periods of the training sessions. Two separate criteria were used for trial sorting: lick rates and correctness. For lick rates, trials were sorted into low/no licking (lowest two histogram bins) or moderate/high licking (all other histogram bins; Fig. 2*B*, top). For correctness, the same trials were separately sorted into chance performance (below or at 75th quantile) or good performance (above 75th quantile; Fig. 2*B*, bottom). This resulted in two separate binary axes to describe the same day-by-trial matrix (Fig. 2*C*, left). Merging these two axes generated a tripartite state category system (Fig. 2*C*, right)—instead of a quadripartite system, as good performance was always associated with moderate/high licking rates. The three state categories were defined as follows: “persistent” moderate/high licking at chance performance; “disengaged,” low/no licking; and “attentive” good performance. This category system was individualized for each mouse.

A decision-tree classifier was then used to determine whether eight nonperformance variables (Fig. 2*D*) could predict these state categories in a trial-wise manner. The goal was to investigate whether motoric and physiological activity fluctuations could indirectly mark task performance metrics. Mouse-specific decision-tree classifiers were trained on the original “persistent,” “disengaged,” and “attentive” labels (see Materials and Methods; Fig. 2*E*). The predictive efficacy of nonperformance variables was then evaluated as illustrated in Figure 2*F*. Specifically, predictions like the one in Figure 2*E* were iterated 100 times using 1–8 nonperformance variables chosen at random. ROC curves were then produced per group of 100 iterations by accumulating their false-positive and true-positive rates.

ROC curves from a representative mouse (Fig. 2*F*) illustrate that the AUC for each state category increased with the number of nonperformance variables, i.e., the more diverse the pool of nonperformance variables, the better the prediction. This increase in AUC was more pronounced for the “persistent” and “attentive” categories, indicating that their predictions benefited the most from the number of nonperformance variables. This effect persisted when generating predictions from across mice (Fig. 2*G*). AUC values were then plotted as a function of the number of nonperformance variables, resulting in asymptotic curves increasing sharply up to three variables, and then less steeply from 4–6 variables before plateauing at 7–8 variables (Fig. 2*H*). The asymptotic pattern was validated statistically through *R*^2^ coefficients and *p* values from an asymptotic exponential model (see Fig. 2*H* and Materials and Methods). This shows which data volumes could be insufficient, optimal, or redundant for predictive accuracy in this study and potentially other similar studies.

To explore the contribution of specific nonperformance variables and provide additional validity, the same analysis was conducted using individual variables per prediction iteration, resulting in Figure 2, *I* and *J*. According to the data, the classifier performed poorly across all single-variable iterations, with the “attentive” category never exceeding an AUC of 0.63. The prestimulus lick rate was the only variable yielding AUC over 0.79, yet these values were limited to the “persistent” and “disengaged” categories and were still lower than the accuracy levels achieved with the multivariable iterations (compare Fig. 2*I*,*J* with Fig. 2*G*,*H*).

Thus, combinations of motor and physiologic activity variables not explicitly manipulated by the experimenter can reflect performance-based state categories, with the “persistent” and “attentive” categories benefiting the most from higher numbers of variables. Importantly, no individual variable was able to predict state accurately, but combinations of them were required. These results were generally replicated when analyzing within each mouse (Extended Data Fig. 2-2).

See Extended Data Figure 2-3 for an analysis comparing within-mouse predictions and between-mouse predictions—the latter showed lower overall accuracy, supporting the individualized task performance framework proposed here. See also Extended Data Figure 2-4 for alternative approaches to state classification, using regression models (GLM and MNR). Observations from these models reinforce the classification analysis proposed here while elucidating specific influences of each nonperformance variable on state prediction. In particular, neocortical MUA power and PSS showed negative MNR *t* statistic values (see Materials and Methods) during disengaged states, consistent with literature associating quiescence with lower MUA and more negative PSS (Vinck et al., 2015; Gao et al., 2017).

Other additional controls are presented in Extended Data Figure 2-5. First, the predictive power of prestimulus licking was quantified, confirming it as the most influential variable in this study. However, prestimulus licking alone was still insufficient to reproduce the asymptotic AUC trends observed in multivariable predictions (Extended Data Fig. 2-5), especially for the attentive state—the most challenging to predict. Second, state predictions were tested using two subsets of variables—physiological/behavioral (wheel, licking, and eye activity) and neurophysiological (LFP and MUA). Physiological/behavioral variables were more influential for state predictions than neurophysiological variables, though the difference was modest (Extended Data Fig. 2-5), supporting the emphasis on unspecific variable combinations as mentioned above. Third, Extended Data Figure 2-5 shows results from different data shuffling methods and trial smoothing window sizes, providing further context for intervariable relationships and the timescale of state changes as studied here.

### Learning-like changes in motor activity within each state

Performance is often measured through response or reward rates. Are there behavioral signs of learning outside performance-specific measures? This question is explored in Figure 3, with a focus on learning-related changes within state categories: for example, is the early-training “persistent” state similar to the late-training “persistent” state, after learning has occurred? These differences were quantified by analyzing more fine-scale aspects of behavior: the peristimulus responses.

Trials were first sorted by motivation state (Fig. 3*A*) and then by go/no-go outcome (Fig. 3*B*), with their chronological order preserved. Infrequent trial combinations, i.e., correct rejections and misses during “persistent,” as well as hits and false alarms during “disengaged,” were not analyzed (see Materials and Methods). Then, within each trial subcategory, training-related changes were analyzed relative to the proportion of training progress, for comparability between trial categories (Fig. 3*C*). For that, each set of trials was divided into the same number of blocks (99 trial blocks), and the peristimulus curves within each block were averaged, resulting in a common training progression scale (Fig. 3*C*). The 99 blocks were then divided into early, mid, and late training, 33 blocks each (Fig. 3*C*).

Peristimulus patterns in licking, wheel, and eye activity (Fig. 3*D*) were analyzed in relation to trial subcategory (Fig. 3*E*, columns of graphs) and training stage (Fig. 3*E*, curve colors) across all mice. Lick rates peaked higher in all hit trials but showed lower peaks with a slower decay as training progressed (Fig. 3*E*, first row; see Table 1 for statistics). Notably, this reduction in peak licking activity over training was observed in both “attentive” hits and “persistent” hits (Fig. 3*E*, first row insets; see Table 1 for statistics, where groups of rows and columns are organized as in Fig. 3*E*). This suggests that licking becomes more efficient with training, even during periods of “persistent” low correctness. This learning-based change in licking behavior would be invisible to conventional correctness metrics, which would rather classify high-activity, low-efficiency periods as “poor performance.”

Signs of learning within “persistent” states can also be observed in forward wheel running. In both “persistent” hits and “persistent” false alarms, the animals acquired a pre- and during-stimulus locomotion pattern, with higher acceleration as training progressed (Fig. 3*E*, second row; Table 1, second group of rows). This suggests that even during “persistent” periods, the animals developed habits, as they learned to anticipate the opportunity to consume water. “Persistent” false alarms additionally showed a steep decrease in wheel speed after stimulus, especially during midlate training (Fig. 3*E*, second row; Table 1, second group of rows). This steep decrease represents a locomotion-halting behavior developed during false alarms as training progressed, which is interpreted here as a motoric sign of error recognition. Also in wheel activity, “attentive” correct rejections (but not misses) changed from modest prestimulus forward running at early training to delayed-onset backward running at midlate training (Fig. 3*E*, inset in the second row). This suggests that animals learned to suppress motor activity during “attentive” correct rejections as training advanced. Therefore, even within the “attentive” category, the animals showed learning-like behavioral changes, reinforcing the notion that learning is quantifiable beyond performance metrics. Conventional correctness metrics would not be able to capture these nuances, as attentive states would be simply classified as “plateau performance.”

Peristimulus pupil diameter and eyelid aperture curves displayed slower and subtle responses to stimuli. These responses were magnified by *Z*-scoring individual curves in relation to their 1 s prestimulus windows, revealing slopes that varied with go/no-go outcomes. Hits and false alarms generally elicited poststimulus descending and ascending slopes, respectively, independent of state (Fig. 3*E*, third and fourth rows; Table 1, third and fourth groups of rows). These patterns could indicate cognitive states reacting to response accuracy, ranging from familiarity to surprise. Correct rejections and misses, on the other hand, evoked curves with less clear or no slopes (Fig. 3*E*, third and fourth rows; Table 1, third and fourth groups of rows), possibly reflecting the lower motricity of these trials. However, when comparing training stages, state-specific patterns emerged. “Attentive” state correct rejections showed an increase in pupil diameter during late training compared with early and midtraining (Fig. 3*E*, third row; Table 1, third group of rows). The opposite was observed in eyelid aperture during “attentive” hits and false alarms, as well as “disengaged” correct rejections and misses (Fig. 3*E*, fourth row; Table 1, fourth group of rows). These results suggest a partial dissociation between state-linked pupillary responses and rodent facial expressions (Dolensek et al., 2020; Le Moëne and Larsson, 2023), here inferred from eyelid aperture.

Overall, head-fixed mice can collaterally acquire stereotyped habits and uninstructed movements as a consequence of behavioral conditioning, consistent with previous studies (Hulsey et al., 2024; Yin et al., 2025). As demonstrated here, this form of collateral learning can be decomposed based on both state and go/no-go outcome, which intersect to form specific peristimulus patterns in licking, wheel and eye activity.

### Learning-related changes in neocortical laminar activity within each state

In Figure 4, signs of learning within each state were also investigated, this time in neocortical laminar electrophysiological activity. The S1 cortex was recorded using a linear silicon probe (64 electrodes, 20 µm apart) oriented perpendicular to the cortical surface, spanning all cortical layers (Fig. 4*A*, left). Here, the 45 topmost channels (0.9 mm) were analyzed, roughly corresponding to the thickness of the S1 cortex. Figure 4*A* (right) exemplifies trial-averaged LFP responses to whisker stimuli, highlighting a prominent voltage response within 100 ms from stimulus onset. Figure 4*B* illustrates the corresponding CSD and MUA power matrices (see Materials and Methods). Within 100 ms poststimulus, CSD showed stratified sinks and sources, and MUA power showed initial inhibition followed by heightened multineuronal activity, especially ∼0.6 mm depth (putative deep layers), similar to a previous study in the visual cortex (Senzai et al., 2019). Individual CSD and MUA matrices, one per stimulus, were then sorted by both state category and trial outcome (Fig. 4*C*, left) and averaged into 99 blocks of trials per category (Fig. 4*C*, right). Trial blocks were subsequently grouped into early-, mid-, and late-training stages, 33 blocks each, as described previously for peristimulus motricity.

10.1523/ENEURO.0417-25.2026.f4-1 Figure 4-1 **S1 cortex responses to whisker stimuli: specification to sensitive laminar depths, followed by state comparisons within training stages. A,** Current-source density (CSD) response strengths at superficial channels (0.20-0.36 mm from brain surface) were averaged and sorted by training stage, motivation state and go/no-go outcome across mice. By removing the laminar depth dimension, this approach facilitated direct comparisons among trial types within each training stage (see Fig. 4*C* methods for trial sorting details). **B,** Sorted trials across three mice (datapoints) are shown as violin distributions, statistically quantified using Friedman’s test (see chi-square and P values). Lettered labels for go/no-go outcomes (H: hits, F: false alarms, C: correct rejections, M: misses) are color-coded by state, indicating pairwise post-hoc differences between trial types (P < 0.01, Tukey’s method). **C-D,** Same as panels A-B, but for multi-unit activity (MUA) response strengths at deeper channels (0.50-0.64 mm). Statistical effects varied with both electrophysiological metric and training stage: stronger for superficial CSD during mid training, and stronger for deep MUA during late training. Furthermore, post-hoc comparisons show, for example, stronger superficial CSD during early-training disengaged misses and stronger deep MUA during late-training attentive correct rejections. This supplement underscores that training stage, motivation state, and go/no-go outcome interact to modulate laminar activity profiles, as mentioned in the main text. Download Figure 4-1, TIF file.

For quantification of trial blocks across mice (*n* = 3 in this analysis), their corresponding CSD and MUA matrices were processed into neocortical depth profiles (Fig. 4*D*,*E*, curve graphs) using the following steps: (1) conversion to absolute values to emphasize response strength irrespective of response direction (e.g., sinks and sources contributed equally to quantification), (2) averaging within the 0–100 ms poststimulus window per channel, and (3) detrending and normalization (see Materials and Methods). This revealed laminar patterns underlying stimulus responses, with features aligning roughly with canonical locations of superficial and deep layers (Adesnik and Naka, 2018).

As shown in Figure 4, *D* and *E*, these laminar features changed in strength from early to late training but in different manners depending on state and go/no-go outcome. In CSD responses (Fig. 4*D*), the most prominent training-related effect was a reduction in signal strength around superficial channels (∼0.2–0.4 mm), especially during stimuli leading to “persistent” hits and false alarms. In MUA responses (Fig. 4*E*), training-related changes were concentrated in deeper channels (>0.5 mm), especially during stimuli leading to “attentive” hits, “attentive” false alarms, and “attentive” correct rejections, as well as during “disengaged” states. More generally, these response peaks around superficial and deep channels were attenuated during “attentive” states across both CSD and MUA responses, regardless of early-, mid-, or late-training stages (Fig. 4*D*,*E*). Notably, the direction of whisker deflection stimuli—caudal for go and rostral for no-go trials—cannot alone explain the results shown in Figure 4, *D* and *E*, as the effect of training on stimulus responses preferably varied with state and trial outcome. For example, training progression effects on CSD were stronger in both “persistent” hits and “disengaged” misses compared with their counterparts in “attentive” states, even though whisker deflections were always caudally oriented in these trials (Fig. 4*D*). Similarly, training progression had little effect on MUA within “persistent” false alarms, while “attentive” false alarms, “attentive” correct rejections, and “disengaged” correct rejections all showed late-training MUA peaks around deeper channels (∼0.5–0.7 mm), even though whisker deflections were always rostrally oriented in these trials (Fig. 4*E*). See Table 2 for statistics.

In summary, S1 laminar activity changes according to the intersection of training progression, motivation states, and go/no-go outcomes, revealing plasticity-like anatomical and functional patterns. Along with the observations on peristimulus motor activity presented earlier, these findings reinforce the multifaceted nature of learning and motivation state fluctuations. See also Extended Data Figure 4-1 for an alternative analysis focused on specific depth segments and trial type differences within each training stage.

## Discussion

This study explored head-fixed mouse behavior and physiology on two overlapping timescales: go/no-go learning between training sessions and motivation state variations within sessions. Through relatively simple analyses like decision-tree classification and time-series comparisons, a continuum linking these timescales emerged. This continuum appeared in task performance metrics (correctness, response rates) and nonperformance metrics (motor and brain activities that changed with training, despite no direct manipulation). The results suggest that nonperformance metrics are flexible across both learning and motivation state, with each animal following a unique learning-motivation trajectory. Therefore, learning and motivation state variations can be tracked holistically from the brain and body, beyond performance metrics such as response rates or conditioning curves. In addition, these variations can be classified per subject, providing insights for individualized behavioral training.

### Go/no-go responses and associated behaviors: state changes over tens of trials

Go/no-go and other behavioral conditioning methods traditionally quantify discrete responses like licking or lever pressing (Guo et al., 2014; Akhlaghpour et al., 2016; Gordon-Fennell et al., 2023). These responses can be monitored over trials or training days to form learning curves, which can be analyzed in terms of stimulus salience or motivational state (Allen et al., 2019; Ceballo et al., 2019). Although invaluable for studying sensory and memory processes, this approach often overlooks behavioral and physiological patterns that are not directly linked to task performance. The present study emphasized a broader (though nonexhaustive) view of motor and physiologic activity surrounding the go/no-go response, from anticipatory wheel running and consummatory licking to laminar LFP responses in S1.

This comprehensive view of task trial behaviors aligns with previous studies (Neske et al., 2019; Salkoff et al., 2020; Cazettes et al., 2021; Matteucci et al., 2022), but here learning was more explicitly examined alongside motivation state fluctuations. During each training session, 500 task trials over an hour captured a decay from high to low responding. This trend featured relatively abrupt state changes, approximately within 50 trials (<5 min), similar to previous studies (Reimer et al., 2014; Vinck et al., 2015; Collins et al., 2023; Hulsey et al., 2024; Yin et al., 2025). To match that temporal scale, lick rates and correctness were here analyzed using 50-trial moving averages (one-trial steps), revealing state changes more clearly. By thresholding these state changes, task trials were then classified into “persistent,” “attentive,” or “disengaged.” Other studies have also converged into tripartite state category systems (Matteucci et al., 2022; Hulsey et al., 2024), typically reflected by a decay from high to moderate to low activity over a training session.

Subsequently, fluctuations in the nonperformance metrics of wheel, eye, and brain activity were observed and hypothesized to reflect underlying states—as represented by both training progression and the state changes described above. However, the fluctuations in nonperformance metrics were subtle, requiring a decision tree to classify states based solely on these metrics. This resulted in accurate predictions, consistent with studies that used other variables for state classification (Salkoff et al., 2020; Zerlaut et al., 2022; Turner et al., 2023). Interestingly, model accuracy depended on individual animal variability, supporting prior evidence of subject-specific learning of uninstructed behaviors (Hulsey et al., 2024), seen here in learning-like changes in peristimulus activity.

Spontaneous reversals from low to high activity were also observed, like a previous study (Yin et al., 2025), with state durations varying from session to session. This suggests that task engagement is not exclusively linked to appetitive motivation, also consistent with previous research (Ortiz et al., 2020). Furthermore, the incidence and duration of “attentive” states varied per animal, and changes in nonperformance variables during “persistent” states were measurable across days. These findings match evidence that nonhuman animals are capable of implicit individual-specific learning (Boogert et al., 2018; Kuchibhotla et al., 2019), again represented here by learning-like changes in motor and physiological activity patterns within poor-performance periods.

It is worth noting that multitrial moving averages like those used here and in a previous study (Matteucci et al., 2022) are more suited for examining state tendencies over tens of trials. This approach contrasts with other previous studies (Ashwood et al., 2022; Hulsey et al., 2024) that focused more on rapid state transitions and strategy switching at single-trial resolution. Both analytical frameworks have their unique contributions: while trial-wise models reveal fast state switching (more relevant to decision-making and task strategies), smoothing over multiple trials captures slower changes in engagement (more relevant to ongoing motivation states). Jointly examining these two temporal dimensions could elucidate how motivation interacts with rapid state transitions to drive decision-making behavior, including in go/no-go paradigms.

### Motor and physiological activity across learning and motivation states

State-related changes in mouse locomotion and licking activity were observable “by eye” in the present experiments. In contrast, changes in mouse facial and brain activity were less apparent and required quantification. Despite these differences in visibility, all variables complemented each other for state classification, with prestimulus licking being the most influential (though still not sufficient) predictor for model accuracy. The interplay of wheel running and pupil dynamics, in particular, has been intensely documented in the past decade due to advances in rodent monitoring. Both wheel running and pupil dynamics are known to accompany cognitive and sensory shifts tied to task demands or locomotor engagement (Erisken et al., 2014; Lee and Margolis, 2016; Mineault et al., 2016; Schriver et al., 2018, 2020; Shimaoka et al., 2018; Neske et al., 2019; Liu et al., 2021; Narasimhan et al., 2023; Hulsey et al., 2024; Yin et al., 2025). These shifts have been primarily linked with fluctuations or phasic activations in cholinergic and monoaminergic neuromodulation, which in turn are known to affect forebrain state, sensory responsiveness, and behavioral flexibility (Reimer et al., 2016; Cazettes et al., 2021). The present study suggests that different manifestations in behavior and physiology—from overt licking patterns to subtle pupil movements—are all complementary markers of both learning and state, which together form a timeline amenable to future studies involving neurotransmitter monitoring. Moreover, the eyelid aperture measurements in this study were not fully redundant with pupil dynamics. In addition to supporting the notion that state-related facial postures are quantifiable in rodents (Dolensek et al., 2020; Le Moëne and Larsson, 2023), the partial nonredundancy between pupil and eyelid movements diversified the dataset used here, reinforcing that states and their neural underpinnings are multifaceted and remain underexplored.

A neocortical depth profile was also examined in relation to learning, state, and whisker stimuli using CSD and MUA analysis. Layer identification, as done in previous works (Senzai et al., 2019), was beyond the scope of this study, limiting circuit-level interpretations on neocortical cell types and stratified innervation patterns (Adesnik and Naka, 2018). Nevertheless, the present analyses reveal anatomical and functional patterns that are potentially informative for future studies. These patterns include heterogeneities along the dorsal–ventral axis of the S1 cortex, with prominent features in CSD and MUA responses undergoing state modulation at consistent anatomical locations, particularly superficial (∼0.2–0.4 mm) and deep channels (∼0.6–0.8 mm). As training progressed, these features changed in opposite directions between CSD and MUA, with superficial CSD peaks being attenuated and deep MUA peaks being strengthened with training. Interestingly, these training-driven changes were heterogeneous across states, with superficial CSD peaks becoming further attenuated during “persistent” and “disengaged” states and deep MUA peaks emerging more clearly during “attentive” and “disengaged” states. In addition, an interaction was observed between training progress and go/no-go outcomes, with deep MUA peaks emerging more prominently than superficial MUA peaks in “attentive” hits, false alarms, and correct rejections, as well as “disengaged” correct rejections and misses. This suggests that stimulus-evoked S1 cortex responses emerge at certain preferred depths, but plasticity-like adjustments occur depending on state, go/no-go response, and training status, consistent with findings from other neocortical areas (Erisken et al., 2014; Neske et al., 2019; Zempeltzi et al., 2020; Matteucci et al., 2022; Morrill et al., 2022). Further investigation is needed combining motivational state, response type, task expertise, and neocortical layers, for example, leveraging trial parsing systems like the one proposed here.

### Other limitations and strengths

Head-fixed behavioral conditioning involves unnatural stimulus–response contingencies. Here, these drawbacks are partly represented by the whisker deflection stimuli, which prevented active whisker palpation, as measured in other studies (Cheung et al., 2019). Other limitations include the bias toward impulsive licking at the beginning of each session, as noted previously (Guo et al., 2014), and the lack of more detailed facial videography to assess emotional states (Dolensek et al., 2020; Le Moëne and Larsson, 2023). Additional confounders include the overlap among anticipatory (pavlovian) licking, task-controlling (operant) licking, and consummatory licking, which can be challenging to disentangle in head fixation studies like the present one, e.g., without operant choice like lick-left or lick-right as studied previously (Guo et al., 2014). Stress from head fixation is also a confounder, as stress is known to affect state and cognitive performance (Kim and Kim, 2023). Finally, experimental challenges led to missing data, particularly in laminar electrophysiology, reducing the sample to three animals in that part of the study.

Despite these issues, both head fixation and the 5 s trial structure induced measurable patterns in motor and physiological activity, reflecting performance variations within sessions and across learning. Additionally, the 5 s trial structure prompted the animals to learn a behavioral “rhythm,” achievable by avoiding “breaks” in that rhythm, i.e., the 10 s punishments during false alarms. With such timings, the animals developed stereotyped uninstructed behaviors (Hulsey et al., 2024; Yin et al., 2025), including bursts of wheel running, which became increasingly aligned with the 5 s trial structure as training progressed. This again indicates learning-like changes in nonperformance variables: an observation facilitated by methods such as the ones used here.

### Practical implications and concluding remarks

Human cognition studies frequently combine performance metrics (e.g., correctness, reaction time) with noninvasive state metrics (e.g., pupil size, heart rate, brain responses; Filipowicz et al., 2020; Burlingham et al., 2022; Luna et al., 2023). The tasks are often learned within minutes, coinciding with state fluctuations affecting attention and perception (Shalev et al., 2019). However, the continuum formed by state fluctuations and longer-term learning is less explored in humans. This mouse study provides insights into that continuum. Task trials were classified using behavioral and physiological variables, revealing learning and state capabilities that varied per individual. This approach could potentially be applied to humans, provided that methodological consistency is maintained between training sessions. This would allow researchers to better characterize the learning and state “style” of each individual, informing interventions like virtual reality training and biofeedback (Faller et al., 2019; Ziegler et al., 2022; Hinss et al., 2023; Meissner et al., 2023). Thus, decomposing behavioral and physiological states, as done here in mice, can be analytically useful, especially if the ecological validity of that decomposition is considered (Krakauer et al., 2017).

## Synthesis

Reviewing Editor: Mihaela Iordanova, Concordia University - Loyola Campus Decisions are customarily a result of the Reviewing Editor and the peer reviewers coming together and discussing their recommendations until a consensus is reached. When revisions are invited, a fact-based synthesis statement explaining their decision and outlining what is needed to prepare a revision will be listed below. The following reviewer(s) agreed to reveal their identity: Paul Masset, Philip Jean-Richard-Dit-Bressel.

The reviewers found the work interesting as it provides a thorough characterization of how the different physiological and neural variables are related to these three behavioral states. Showing that variables not directly related to task performance (defined as correct/error) convey extensive information on the behavioral state and predict task performance would be value to the field. Nevertheless the reviewers outlined a number of concerns some overlapping that need to be addressed in a revision.

1. Both reviewers had comments related to variable choice.

a. The key claim is that more than one non-performance variable meaningfully improves state predictions, but this is based on randomly selecting from a set number of non-performance variables. Almost all the predictive power comes from pre-stimulus licking, which is arguably the least interesting of the variables used to predict post-stimulus licking. The proposed weakness of single variables was because this analysis randomly selected from all variables and averaged across results. I surmise the asymptotic AUC gain with increasing variables was simply because there was an increasing chance that pre-stimulus licking was included in the decision tree. If pre-stimulus was always included in the decision-tree, I question whether there would be an asymptotic trend. If authors wish to maintain the key claim, they should show whether prediction accuracy using a tree with [n+1 variables] was a significant improvement over the best [n variables] tree, preferably while accounting for the increased complexity of the n+1 tree. This is the more principled approach, given the small number of variables in play.

b. The pre-stimulus licking rate appears to be the strongest signal, but it could be argued to be more task-related than the others as reward consumption requires licking. How do the analysis on Fig 2F,G look like if pre-stimulus licking is omitted?

c. The analysis presented in Fig 2E-J is interesting as it quantifies how much the non-task variables can predict the behavioral state of the animal. However, the types of variables used for the prediction is very different. It would be interesting to perform the analysis independently for the physiological/behavioural variables and for the neurophysiological variables. Theis would provide a more independent assessment of how much the neural activity in S1 is predictive of behavioral state.

d. Impulsive trials are grouped into one category in which the licking per trial is high, but the performance is low. Interestingly in these trials, the CSD analysis identifies a difference across laminar depth with stages of training. One possible concern is that these trials could be quite different from the point of view of the animal. Early in training, the animal has never reached high accuracy so the licking might be more due to exploration rather than true impulsivity. Later in training, these could be due to true impulsivity. Beyond the CSD analysis, would some of the non-task variables able to predict task accuracy (regression rather than classification) or training progress? Considering the impulsive category is simply selected based on low accuracy-high licking criteria, it could hide additional subtleties that this analysis could uncover. .

2. The decision tree classifier was not tested against a truly shuffled dataset. It is unclear whether randomizing timestamps per variable would remove the capacity for the classifier to predict the state. Ideally, the statistical significance of the classifier against this truly shuffled comparison would be quantified, e.g. via permutation test.

3. It was not clear to me how reward was delivered, and thus how licks could be operant vs. consummatory. This needs to be explained in Methods and added to Figure 1D (if there is set time that reward is delivered/available). Even if there were licks that triggered subsequent reward delivery, and not just consumption, the claim that licking behavior was "operant" is debatable (see doi: 10.3758/BF03331699). Licking to the go cue but not no-go cue can be easily interpreted in a Pavlovian framework. Licking behavior in this study should be framed more carefully. The reference to "preparatory action habits" (line 586) should be removed. Subjects' wheel running can also be framed in this Pavlovian framework.

4. Additional justification for the for the 50 trial window is needed.

a. The authors have categorized trials into behavioral states using a simple thresholding measure across licking and accuracy, measured using a large sliding window (50 trials). Are the results robust to changes in the size of the window? Alternatively, approaches like GLM-HMM (Ashwood et al., Nat Neuro, 2022) could provide a more principled to classify behavioral state throughout the task. Have they been attempted on the data? They should at least be cited in the discussion of alternative approaches

b. This window strikes me as quite long, representing a minimum of 250sec, which is much longer than common notions of how rapidly arousal states can fluctuate. Relatedly, were all trials in the moving window weighted equally (i.e distal trials influenced the state categorisation as much as the current or more proximal trials)? A weighting function (e.g. Gaussian) might have been preferable. The impact of these choices should be considered in Discussion.

5. Authors do not provide a rationale for the cut-offs used to classify states (2nd percentile lick rate, 75th percentile correctness). Applying a data-driven clustering algorithm across subjects would have been a less arbitrary way of determining states. Determining by a fixed percentile per subject risks grouping different kinds of trials, as some animals might be generally better/worse at the task than others (ie 75th percentile represents a sensible cut-off for one subject but not others). Providing supplementary data per subject (e.g. histograms, as provided in Figure 2B) in Supplementary Materials would help readers evaluate the appropriateness of these cut-offs.

6. Can one subject's decision tree be used to accurately predict another mouse's state? This would speak to the generalizability of relationships between behaviors. Based on supplementary materials, it seems non-performance variables were differentially predictive across subjects. Did these decision tree differences between subjects predict individual differences in task behavior? Implications of these inter-individual differences should be discussed.

7. The reason for excluding mice from LFP analysis was not adequately explained. Exclusion criteria must be detailed in Methods.

8. S1 LFP results section refers to differences between LFP across trial types (e.g. lines 646-649). However, Table 2 only reports [training stage x depth] analyses. There is no comparison between arousal state and/or trial types. Significant vs null effects per trial type is not sufficient evidence for a meaningful difference. A direct comparison with trial types as a factor should be reported to support the argument that laminar features depended on arousal state and go/no-go outcome.

Minor

1. Fig 1E. Single animal performance could be shown as thin line to provide a better description of variability across animals.

2. Which 99 trials were chosen for early/middle/late analyses? The first 33, middle 33 and last 33? This should be detailed in Methods.

3. Just to clarify, for trials at the beginning and end of each session, did the moving average only draw from available trials for that session, and thus averaged across a smaller window?

4. line 603: "independent on" should be "independent of"

## Author Response

Dear Editor and Reviewers, we appreciate the synthesis of reviews and the specific reviewer comments. Our responses are in blue font, including "action taken" statements in italic bold font and yellow highlights to indicate edits or emphases in the text and figures.

We have added five Extended-Data figures, each accompanied by manuscript edits and detailed figure legends. The order of these ED figures matches their sequence of appearance within the manuscript, not the sequence in this response document.

Computational Neuroscience Model Code Accessibility Comments for Author (Required): Please include a data and code availability statement along with a link where the code can be found.

Response: To address this requirement, we have added a "Code accessibility" statement at the end of the Methods section (lines 568-572).

Please note that this is an experimental (not computational) neuroscience study. We interpret this requirement as referring to our state classification code involving decision trees - this is the nearest equivalent to computational neuroscience in our study. Thus, the newly added "Code accessibility" statement contains a link to that code.

Also note that we typically deposit code to the laboratory GitHub page. To preserve author anonymity during the next round of double-blind reviews, the link was redacted from the marked-up version of the revised manuscript. This action follows guidance from eNeuro's editorial staff, with whom we consulted via email.

Synthesis Statement for Author (Required):

The reviewers found the work interesting as it provides a thorough characterization of how the different physiological and neural variables are related to these three behavioral states. Showing that variables not directly related to task performance (defined as correct/error) convey extensive information on the behavioral state and predict task performance would be value to the field. Nevertheless the reviewers had a number of conceptual and methodological concerns that have been outlined below and need to be addressed in a revision.

Response: Thank you. This is an accurate synthesis of our motivation in this study: using videographic and physiological monitoring to extend the characterization of task performance fluctuations beyond correctness metrics.

1. Both reviewers had comments related to variable choice. a. The key claim is that more than one non-performance variable meaningfully improves state predictions, but this is based on randomly selecting from a set number of non-performance variables. Almost all the predictive power comes from pre-stimulus licking, which is arguably the least interesting of the variables used to predict post-stimulus licking. The proposed weakness of single variables was because this analysis randomly selected from all variables and averaged across results. I surmise the asymptotic AUC gain with increasing variables was simply because there was an increasing chance that pre-stimulus licking was included in the decision tree. If pre-stimulus was always included in the decision-tree, I question whether there would be an asymptotic trend. If authors wish to maintain the key claim, they should show whether prediction accuracy using a tree with [n+1 variables] was a significant improvement over the best [n variables] tree, preferably while accounting for the increased complexity of the n+1 tree. This is the more principled approach, given the small number of variables in play. b. The pre-stimulus licking rate appears to be the strongest signal, but it could be argued to be more task-related than the others as reward consumption requires licking. How do the analysis on Fig 2F,G look like if pre-stimulus licking is omitted? Response to comments 1a and 1b: We have created a new figure (ED Fig. 2-5A) and referenced it in the revised Methods (lines 463-467) and Results (lines 670-674). Below we contextualize these additions.

Reviewers suggested that the asymptotic trends (Fig. 2H) might be explained solely by one variable: pre-stimulus licking. In contrast, we had claimed in the original manuscript that "no individual variable was able to predict state accurately, but combinations of them were required" (lines 657-658).

Our new analysis (ED Fig. 2-5A) further supports our claim. It is true that pre-stimulus licking is the most influential predictor in our data, which we had noted in the original manuscript (lines 650-654). However, the new analysis shows that the asymptotic trends remain significant in both scenarios: when pre-stimulus licking is always omitted and always included (ED Fig. 2-5A).

There are additional nuances worth noting, and we thank the reviewers for the opportunity to elaborate on them. When pre-stimulus licking is always omitted (ED Fig. 2-5A), the asymptotes remain strong but their plateaus decrease slightly, reaching 0.86 AUC for the persistent/attentive states compared to 0.89 AUC in the main analysis (Fig. 2H) - a modest 0.03 reduction. Conversely, when pre-stimulus licking is always included (ED Fig. 2-5A), the asymptotic trends become weaker - though still significant - and AUC values reach the same high levels as the main analysis (Fig. 2H).

In other words, repeatedly omitting pre-stimulus licking weakens the overall accuracy but preserves the asymptotic curve shapes, while repeatedly including pre-stimulus licking does the opposite. These observations add helpful nuance, but are secondary to the main story, in our view. Hence, we decided to present these new findings as supplementary (ED Fig. 2-5A).

Also important: the "attentive" state was overlooked in the reviewer comments. Although pre-stimulus licking is indeed a strong predictor, its influence is mostly limited to the persistent and disengaged states. This can be visualized in the original data (Fig. 2I,J and ED Fig. 2-2D-F): pre-stimulus licking alone was not sufficient to accurately predict the attentive state. The new analysis further clarifies this dissociation: the asymptotic trends are equally strong for persistent and attentive states when pre-stimulus licking is always omitted, but stronger for the attentive state when pre-stimulus licking is always included (ED Fig. 2-5A).

In summary, by repeatedly omitting pre-stimulus licking we provide further support for one of our original statements: "This shows which data volumes could be insufficient, optimal or redundant for predictive accuracy in this study and potentially other similar studies" (lines 645-647). In turn, by repeatedly including pre-stimulus licking, we further clarify the role of this variable not only for overall model accuracy, but also state differentiation. c. The analysis presented in Fig 2E-J is interesting as it quantifies how much the non-task variables can predict the behavioral state of the animal. However, the types of variables used for the prediction is very different. It would be interesting to perform the analysis independently for the physiological/behavioural variables and for the neurophysiological variables. Theis would provide a more independent assessment of how much the neural activity in S1 is predictive of behavioral state.

Response to comment 1c: It is a great idea to separately test these two subsets of variables (physiological/behavioral and neurophysiological). We have expanded the new supplement mentioned above to include such analysis (ED Fig. 2-5B) and referenced it in the revised Methods (lines 467-470) and Results (lines 674-679).

As shown in ED Fig. 2-5B, predictive power was slightly stronger for the "physiology/behavior subset" compared to the "neurophysiology" subset. Since both subsets are composed of four variables, we can rule out variable imbalance as confounder. Thus, we can interpret that most of the predictive power comes from the subset of physiological/behavioral metrics, especially (but not exclusively due to) pre-stimulus licking, as explained in the previous point.

However, there was only a small difference between the two subsets: the attentive state, i.e., the most challenging to predict, plateaued at 0.84 AUC for the physiology/behavior subset, and 0.82 AUC for the neurophysiology subset (ED Fig. 2-5B). Furthermore, the asymptotic trends reached P values of 0.03 or less in all cases (ED Fig. 2-5B).

We interpret these asymptote P values cautiously given the lower number of data points per asymptote (four compared to eight in the main analysis). This aligns with our main proposal: combinations of four or more variables - whether behavioral, physiological, or neurophysiological - can be sufficient for predicting learning and intra-task states in this and potentially other similar experiments. d. Impulsive trials are grouped into one category in which the licking per trial is high, but the performance is low. Interestingly in these trials, the CSD analysis identifies a difference across laminar depth with stages of training. One possible concern is that these trials could be quite different from the point of view of the animal. Early in training, the animal has never reached high accuracy so the licking might be more due to exploration rather than true impulsivity. Later in training, these could be due to true impulsivity.

Response to comment 1d (part A): In response to the reviewer's concern regarding our use of the term "impulsive", we have renamed "impulsive" to "persistent" throughout the manuscript and figures. "Persistent" is a more objective term, agnostic to the underlying process: whether exploration, impulsivity, or a combination of both. This change aligns with terminology adopted by another research group, which we now cite (Parent et al., 2015 - doi.org/10.3389/fnint.2015.00023; Amarante et al., 2017 - doi.org/10.1523/JNEUROSCI.1965-17.2017).

We would like to emphasize certain methodological details, which were present in the original submission and have been edited for further clarity (lines 111-121). First, water deprivation started one week prior to go/no-go training. Second, mice were pre-trained to lick during two daily acclimation sessions, also before go/no-go training. High lick rates were evident within minutes of the first water administration, persisting for approximately 30 min in each acclimation session. Therefore, the mice were already familiar with the lick spout before go/no-go training.

Given this context, we continue to consider impulsivity as the predominant driver of high-licking periods, regardless of training stage. However, we do not have quantitative evidence for this, as behavioral activity during the acclimation sessions was not recorded. Rather, water drop delivery by the device was initiated "manually" during these sessions, using a graphical user interface (Bpod). We have added these details to the Methods (lines 114-116). With these clarifications in mind, we believe that renaming the high-licking state to "persistent" is a straightforward solution.

Beyond the CSD analysis, would some of the non-task variables able to predict task accuracy (regression rather than classification) or training progress? Considering the impulsive category is simply selected based on low accuracy-high licking criteria, it could hide additional subtleties that this analysis could uncover. .

Response to comment 1d (part B): Regression analysis is indeed an option we had overlooked. To address this, we have created a new supplementary figure (ED Fig. 2-4) and amended the Methods (lines 447-462) and Results (lines 663-669).

The new figure begins with Generalized Linear Models (GLM; ED Fig. 2-4A). Our eight non-performance predictors are depicted as violin distributions in ED Fig. 2-4A, each violin populated with GLM t-values (i.e., the data points). These t-values were calculated using leave-one-predictor-out GLM iterations, where one predictor is omitted per iteration. This process was then repeated across mice, and the resulting t-stats were cumulatively plotted, forming the violin distributions in ED Fig. 2-4A. We used correctness percentage as the response variable, thus testing the ability of these GLMs to predict task accuracy independent of state categories. By doing this, we jointly address the reviewers' interest in task accuracy and in alternatives to state classification (both this comment and comment #4 below).

Pre-stimulus multi-unit activity (MUA) power emerged as the first new subtlety in this new GLM analysis (ED Fig. 2-4A). Other than pre-stimulus licking, i.e., the powerful predictor discussed above, pre-stimulus MUA power was the only variable to significantly differ from shuffled-data controls. The direction of such effect was also informative: t-values for pre-stimulus MUA power were significantly higher than shuffled-data controls, suggesting a positive relationship with task expertise. Conversely, t-values for pre-stimulus licking were significantly lower than shuffled-data controls, suggesting a negative relationship with task expertise.

However, we reacted to these results with skepticism. Since licking activity during attentive states was either high or moderate (Fig. 2C), a negative relationship between pre-stimulus licking and task accuracy seemed counterintuitive to us. One possible confound was that chance-level correctness (~50%) does not distinguish between task disengagement and persistent licking, raising the possibility that disengagement biased the pre-stimulus licking predictor toward negative t-values. To resolve this, we have expanded this analysis to include a multinomial regression approach (ED Fig. 2-4B-C). Unlike GLM, multinomial regression is better suited to predict categorical variables with three or more categories, for example the three states of our main study. Thus, we have further explored the data using leave-one-out multinomial regression iterations. The resulting t-values were then parsed by state category (ED Fig. 2-4B-C).

Interestingly, multinomial regression iterations "decomposed" the GLM results by states (ED Fig. 2-4B-C), clarifying the confounders mentioned above. Specifically: (1) task disengagement was indeed the primary factor driving the negative t-value bias for pre-stimulus licking in the GLM analysis; and (2) attentive task performance was indeed the main contributor to the positive t-value bias for pre-stimulus MUA power in the GLM analysis. Also interestingly, yet more subtleties were revealed by these multinomial regressions, including negatively biased t-values for all electrophysiological metrics during disengaged states. This is consistent with literature associating quiescence with lower MUA and steeper (more negative) power spectrum slopes (PSS) (e.g., Vinck et al., 2015 - doi.org/10.1016/j.neuron.2015.03.028; Gao et al., 2017 - doi.org/10.1016/j.neuroimage.2017.06.078). These new analyses are mentioned in the revised manuscript (lines 447-462 and 663-669) and ED Fig. 2-4 figure legend.

We thank the reviewer(s) for motivating us to further explore our data using regression approaches. However, note that ED Fig. 2-4 is only conclusive due to a combination of correctness predictions (GLM) and state predictions (multinomial), favoring the scope we had chosen for this study, focused on state categories. Thus, although these new regression results are interesting, we consider them supplementary to the main story - that behavioral and physiological variables (1) collectively predict learning and intra-task states, and (2) show plasticity within these states over the days of training.

2. The decision tree classifier was not tested against a truly shuffled dataset. It is unclear whether randomizing timestamps per variable would remove the capacity for the classifier to predict the state. Ideally, the statistical significance of the classifier against this truly shuffled comparison would be quantified, e.g. via permutation test.

Response to comment 2: Agreed. Statistical comparisons against fully shuffled data have been carried out (ED Fig. 2-5C) and contextualized in the manuscript (lines 470-473 and 679-681), enriching our study with controls.

In this new analysis (ED Fig. 2-5C), we used the same iteration loops as in the asymptotic AUC gain analysis (Fig. 2G), where shuffling had originally been restricted to the temporal sequence of task trials (please see lines 407-413, which now contains additional details on trial shuffling). However, for ED Fig. 2-5C the predictor tables were fully shuffled at each iteration, both across task trials (rows) and variables (columns). AUC values from row-shuffled vs. fully shuffled data were then plotted as violin distributions and compared along the "number of variables" x-axis: the same x-axis as Fig. 2H.

As shown in ED Fig. 2-5C, all AUC values from fully shuffled iterations were concentrated around chance levels (0.5), and the statistical differences between iteration groups (row-shuffled vs. fully shuffled) were non-surprisingly robust (Friedman's chi-sq(15) > 4809). Thus, together with the preceding figure panels, ED Fig. 2-5C further supports our approach to state prediction using pools of non-performance variables. We appreciate this and all other related suggestions, which resulted in a supplement integrating various controls.

3. It was not clear to me how reward was delivered, and thus how licks could be operant vs. consummatory. This needs to be explained in Methods and added to Figure 1D (if there is set time that reward is delivered/available). Even if there were licks that triggered subsequent reward delivery, and not just consumption, the claim that licking behavior was "operant" is debatable (see doi: 10.3758/BF03331699). Licking to the go cue but not no-go cue can be easily interpreted in a Pavlovian framework. Licking behavior in this study should be framed more carefully. The reference to "preparatory action habits" (line 586) should be removed. Subjects' wheel running can also be framed in this Pavlovian framework.

Response to comment 3: We acknowledge that our explanation on water delivery was insufficient. Please, see the edits in lines 100-106 for more details on hardware setup (solenoid valve, plastic tubing, lick spout) and lines 169-173 for clarification on trial programming, including how rewards were timed within the 1.8 s answer period. We initially thought the term "answer period" was clear enough to describe reward timing, but now we realize that that assumption was likely incorrect, given this comment. Fig. 1 legend has been updated accordingly to reflect these changes.

For the second part of this comment, we have deleted all instances of "operant" and made minor contextual adjustments where needed. More objective terms like "go/no-go responses" or "task correctness" are now used instead, in the same vein of replacing "impulsive" by "persistent", as discussed above (comment #1d). Also, "preparatory action habits" has been simplified into "habits" (line 714), further contributing to the overall objectivity of the revised manuscript.

On a more conceptual note, we would like to highlight the following clarifying edit (lines 169-173): "Water rewards or false-alarm extensions were triggered just once per trial, upon detection of the first lick anytime within the 1.8 s answer period (Fig. 1D). (...) Further licking in that trial, whether during or after water consumption, did not trigger additional water delivery." In our view, this single water-triggering event (first lick within the answer period) still fits the classical definition of an operant response, i.e., action-outcome, as opposed to Pavlovian stimulus-outcome. However, it is also true that licking and wheel activity occurred before whisker stimuli, which aligns more with Pavlovian "anticipatory conditioned response", as described in the reference cited by the reviewer (Patten &Rudy, 1967). Disentangling operant and Pavlovian components can indeed be difficult in mouse head fixation tasks like ours, since licking is both operant and consummatory, unlike in paradigms where operant behaviors are more distinct (e.g., rat lever pressing in Skinner boxes). Furthermore, design parameters in head fixation setups (e.g., distance between mouth and lickspout, lick-left/lick-right choice) can influence lick timing and compulsivity, as noted in a review that we cite (Guo et al., 2014). We have added a brief amendment about these confounders to the Discussion (lines 919-923).

4. Additional justification for the for the 50 trial window is needed. a. The authors have categorized trials into behavioral states using a simple thresholding measure across licking and accuracy, measured using a large sliding window (50 trials). Are the results robust to changes in the size of the window? Alternatively, approaches like GLM-HMM (Ashwood et al., Nat Neuro, 2022) could provide a more principled to classify behavioral state throughout the task. Have they been attempted on the data? They should at least be cited in the discussion of alternative approaches b. This window strikes me as quite long, representing a minimum of 250sec, which is much longer than common notions of how rapidly arousal states can fluctuate. Relatedly, were all trials in the moving window weighted equally (i.e distal trials influenced the state categorisation as much as the current or more proximal trials)? A weighting function (e.g. Gaussian) might have been preferable. The impact of these choices should be considered in Discussion.

Response to comments 4a and 4b: We have addressed items 4a and 4b both quantitatively (see ED Fig. 2-5D) and textually in the revised manuscript (lines 473-475, 679-681, 854-862), like our earlier response to items 1a and 1b. Please, see elaboration below.

We were not previously familiar with Ashwood et al. (2022). Thanks to this comment, we read their impressive paper, learned about its scope, and included a citation in the Discussion (line 854-862). Their study examines the limitations of standard task performance metrics (e.g., sigmoidal psychometric functions) in differentiating underlying decision-making strategies. The authors use trial-by-trial temporal resolution in their analysis, unlike the longer-timescale resolution used in our work. Also, the authors employ Hidden Markov Models (HMM) to investigate state switching, similar to another study cited in our original and revised manuscript (Hulsey et al., 2024; doi: 10.1016/j.celrep.2024.113709).

However, we would like to point out that long-timescale windowing is also present in the literature. Matteucci et al. (2022; doi: 10.1016/j.neuron.2022.09.032) is a head-fixed mouse study that we cite in our original and revised manuscript. Similar to our work, the authors used "smoothed lick probabilities over a sliding window of 61 consecutive trials" (quoted from their STAR Methods) and recorded task performance for more than 300 trials, thus allowing for spontaneous variations in task engagement. As a result, the authors found licking probability curves very similar to ours. Please, see Figs. 5A, 6B and S5 of Matteucci et al. (2022) and compare to our data in Fig. 2A-C and the top plots of ED Fig. 1-2. We welcome this opportunity to highlight Matteucci et al. (2022), because their publication is contemporary to our data collection period, and yielded converging state fluctuation results, as we had noted in our original manuscript (lines 833-835).

Therefore, different timescales and analytical approaches - whether trial-by-trial state switching or multi-trial state fluctuation - can offer unique contributions to the investigation of learning and intra-task performance variations, as now noted in the revised manuscript (lines 854-862). Other minor changes were made throughout the Discussion section to better accommodate this additional note, including the use of "state changes" instead of "state transitions". We now recognize that "state transitions" is more appropriate for the HMM analyses cited here and in the manuscript (Ashwood et al., 2022; Hulsey et al., 2024).

Another nuance within this topic is, more simply, a matter of terminology. The reviewers' comments prompted us to reflect on the appropriateness of the terms "arousal" and "alertness" for our ~50-trial timescale, given that arousal fluctuations are indeed commonly seen to occur on much shorter timescales (seconds or a few trials). Based on this, we have replaced the terms "alertness" and "arousal" by "motivation" (or more simply: "state") throughout the manuscript, including the title. This aligns with the emphasis that Matteucci et al. (2022) placed on "motivation" as a driving factor in their findings, and also our effort to make our text as objective and cautious as possible (see several responses above).

To substantiate this theoretical discussion with data, we have added a supplement (ED Fig. 2-5D) testing shorter trial window sizes and their effects on the same ROC-AUC curves as our main analyses. Shorter smoothing windows led to poorer predictions (lower asymptotic plateaus) for motivation states, i.e., those occurring on the timescale of tens of minutes, especially the "attentive" state. Shorter windows could have indeed been more appropriate for second- or minute-scale states, e.g., arousal: the term we no longer use thanks to this comment. This additional analysis (ED Fig. 2-5D) provides further controls for Fig. 2, consistent with several responses above.

Finally, we have edited the Methods (lines 337-342) and made several minor changes throughout the manuscript to clarify that: (1) yes, all trials were weighted equally in each windowing step; and (2) windows progressed in 1-trial steps. This means that no artificial boundaries between adjacent 50-trial windows were introduced, ensuring a continuous representation of motivation state fluctuations.

5. Authors do not provide a rationale for the cut-offs used to classify states (2nd percentile lick rate, 75th percentile correctness). Applying a data-driven clustering algorithm across subjects would have been a less arbitrary way of determining states. Determining by a fixed percentile per subject risks grouping different kinds of trials, as some animals might be generally better/worse at the task than others (ie 75th percentile represents a sensible cut-off for one subject but not others). Providing supplementary data per subject (e.g. histograms, as provided in Figure 2B) in Supplementary Materials would help readers evaluate the appropriateness of these cut-offs.

Response to comment 5: Agreed. We have created a new supplement (ED Fig. 2-1) showing histograms from all mice, not just the representative mouse originally shown in Fig. 2B. Fig. 2B has been adapted to reflect the new supplement, including a correction to the task performance histogram. The illustrated cutoff was outdated in the first version - it was lower (i.e., less rigorous) than the cutoff used for quantification. This mistake was confined to the qualitative illustration in Fig. 2B and is now resolved.

In addition, we have included our rationale for the cutoffs in the revised Methods (lines 362-380). At first submission, we considered these details potentially distracting. However, the reviewer's comment made us realize their importance for specialized readers.

These textual edits include another correction: the "low/no licking" trials that characterized disengaged states consisted of the two lowest histogram bins, not the 1st and 2nd percentiles. We apologize for the confusion; please see the correction in line 343. For the correctness analysis, on the other hand, we did use distribution parameters, i.e., quantiles. For further clarity on this, we have replaced correctness "percentiles" with "quantiles" throughout the manuscript, to avoid confusion with correctness "percentages".

6. Can one subject's decision tree be used to accurately predict another mouse's state? This would speak to the generalizability of relationships between behaviors. Based on supplementary materials, it seems non-performance variables were differentially predictive across subjects. Did these decision tree differences between subjects predict individual differences in task behavior? Implications of these inter-individual differences should be discussed.

Response to comment 6: This is another great idea for a supplement. We have created a new figure (ED Fig. 2-3) showing a comprehensive ROC and AUC analysis from all predictor-predicted mouse pairs. All possible mouse pairs were tested, totaling 20 predictor-predicted pairs, 100 decision-tree iterations each. We also did the same analysis for within-mouse predictions like we did in the original manuscript, for control. This effort resulted in ED Fig. 2-3, which contains: (1) a square grid of ROC subplots, with predictor mice in columns and predicted mice in rows; and (2) aggregated AUC values comparing between-mouse predictions and within-mouse predictions - the latter are represented by the diagonal of the ROC grid (ED Fig. 2-3). All non-performance variables were included in this analysis, for comparability with the maximum possible predictive accuracy originally shown in the main manuscript, i.e., the endpoints of the asymptotic plateaus (Fig 2H).

The interesting new observation from ED Fig. 2-3 is that between-mouse predictions yielded reasonably accurate results in aggregate (median AUC of 0.7-0.9). However, this level of accuracy was limited to the persistent and disengaged state categories. And even in these less-challenging states (persistent and disengaged) between-mouse predictions were still less accurate and more variable compared to within-mouse predictions, whose AUC values were more concentrated around 0.9. More importantly, the attentive state showed the strongest statistical difference: AUC values from between-mouse predictions varied around a median of 0.5-0.6, never surpassing 0.75.

In summary, ED Fig. 2-3 clarifies that yes: there is some generalizability from one mouse to another, but not to the same accuracy levels of within-mouse predictions - where the classifier is trained to predict unseen data from the same mouse. Furthermore, this generalizability is state-specific, with the attentive category being especially challenging to predict in this new analysis. These findings are consistent with points made in the original manuscript regarding state-specific predictability (lines 655-660) and individualized task performance analysis (lines 70-72, 810-811, 940-953). Therefore, like previous supplements, ED Fig. 2-3 does not change our main story, though it adds useful nuance to it. We have amended the Methods (lines 438-447) and Results (lines 661-663) to accommodate this new analysis.

7. The reason for excluding mice from LFP analysis was not adequately explained. Exclusion criteria must be detailed in Methods.

Response to comment 7: Thank you for pointing this out. We have added more explicit clarification in the Methods (lines 544-552).

8. S1 LFP results section refers to differences between LFP across trial types (e.g. lines 646-649). However, Table 2 only reports [training stage x depth] analyses. There is no comparison between arousal state and/or trial types. Significant vs null effects per trial type is not sufficient evidence for a meaningful difference. A direct comparison with trial types as a factor should be reported to support the argument that laminar features depended on arousal state and go/no-go outcome.

Response to comment 8: We have created a new figure (ED Fig. 4-1) analyzing trial types within each training stage, rather than the inverse approach used in the main figure (Fig. 4, training states within each trial type). Statistical tests are shown alongside their corresponding subplots. We have included citations to this new figure in the manuscript (lines 537-543 and 796-797) and Fig. 4 legend.

For this new figure (ED Fig. 4-1), we focused on the laminar depth features that exhibited the most prominent change with training stage and trial type, as originally reported in Fig. 4 and retained in the main manuscript. These features are: superficial CSD channels (0.20-0.36 mm) and deeper MUA channels (0.50-0.64 mm). By averaging data within these key channel groups, we were able to eliminate the laminar depth dimension in this analysis, facilitating direct comparisons among all eight trial types - here displayed as violin plots (ED Fig. 4-1).

As shown in ED Fig. 4-1, overall effects (Friedman's chi-square) were stronger for superficial CSD during mid training, and stronger for deep MUA during late training. This complements our suggestion that training stage, motivation state and go/no-go outcome interact to modulate laminar activity patterns (lines 792-794, 900-907). In turn, post-hoc comparisons (color-coded letters above the violins) indicate significant differences (P <0.01) between trial types, including stronger superficial CSD during early-training disengaged misses and stronger deep MUA during late-training attentive correct rejections.

ED Fig. 4-1 provides valuable context, as pointed out by the reviewer. However, analyzing training effects within trial types, as done in the main figure (Fig. 4), remains more closely aligned with our interest in learning-related changes within trial types, including "poor-performance" ones, like hits during persistent licking states.

Minor 1. Fig 1E. Single animal performance could be shown as thin line to provide a better description of variability across animals.

Agreed. Please, see new version of Fig. 1E. We have also made minor adaptations to Fig. 1F to reflect the new supplement with individual mouse curves (ED Fig. 2-1) and to replace "arousal" by "motivation".

2. Which 99 trials were chosen for early/middle/late analyses? The first 33, middle 33 and last 33? This should be detailed in Methods.

Correct: the first, middle and last 33 blocks of trials were quantified as early, middle and late training, respectively. Please, see minor edits in the Methods (lines 494).

3. Just to clarify, for trials at the beginning and end of each session, did the moving average only draw from available trials for that session, and thus averaged across a smaller window? This is also correct. According to the MATLAB Help Center page for the movmean function: "The window size is automatically truncated at the endpoints when there are not enough elements to fill the window. When the window is truncated, the average is taken over only the elements that fill the window." Of note, moving averages were applied separately to each training day before constructing the predictor tables. Therefore, endpoint truncation occurred independently within each training day. We have added this clarification to the Methods (lines 341-342).

4. line 603: "independent on" should be "independent of" Done (line 731). Thank you.
