# Supplementary figures and images for: Learning and Motivation State Fluctuations from Motoric and Neurophysiologic Metrics during a Somatosensory Task in Mice
Source: eNeuro. 2026 May 26;13(5):ENEURO.0417-25.2026. doi: 10.1523/ENEURO.0417-25.2026 (PMC13220975; doi:10.1523/ENEURO.0417-25.2026)

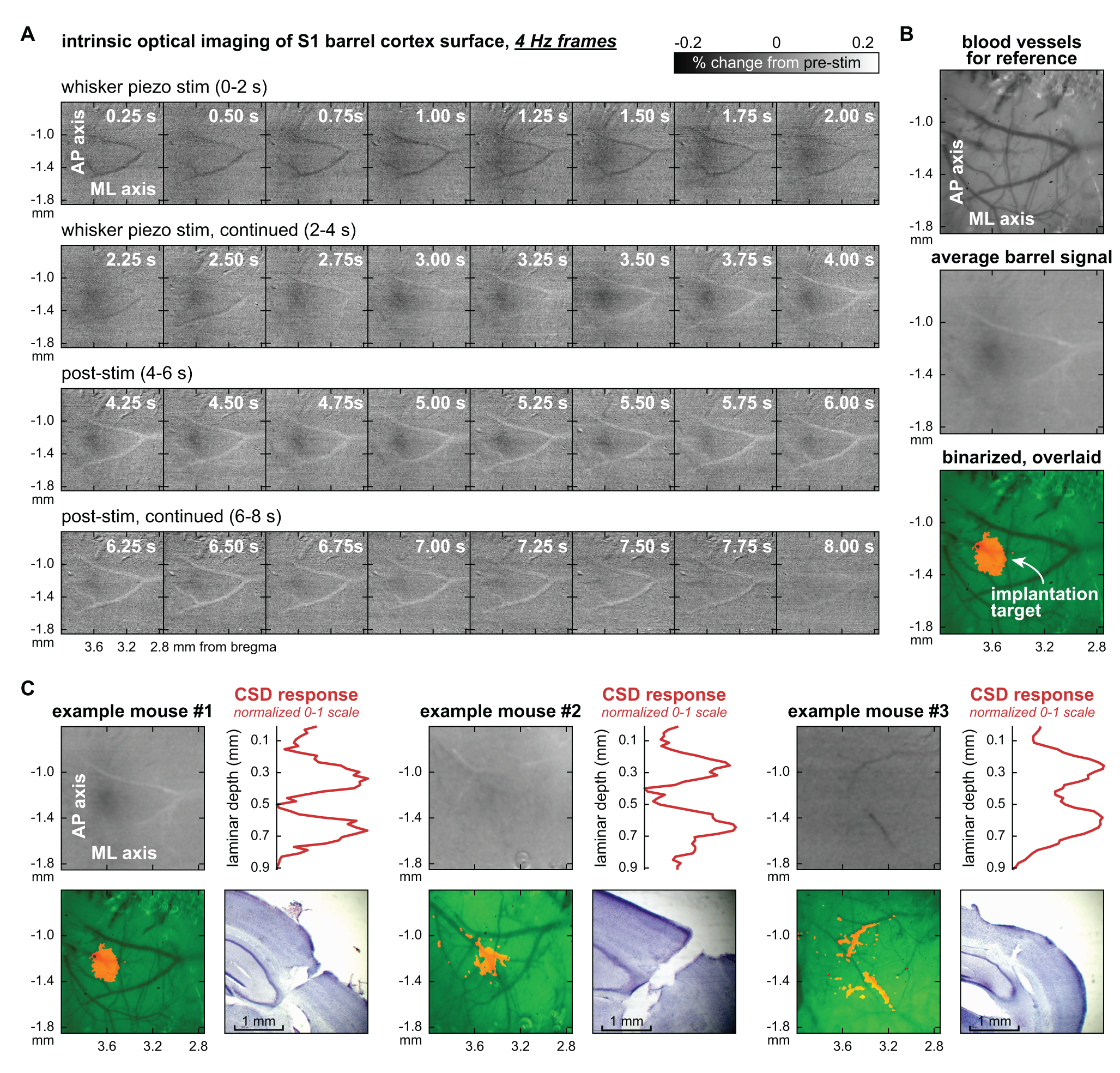

Supplement: Figure 1-1 — Intrinsic optical imaging maps for further specification of S1 cortex implantation coordinates. A, Trial-averaged images from a representative mouse showing surface hemodynamics during and after piezoelectric stimulation of a whisker pair. A thinned-skull window (see coordinates in millimeters) was imaged under high-power red light (630 nm) at 4 Hz for 12 s: 4 s before (baseline, not shown), 4 s during and 4 s after stimulation. Parenchymal and blood vessel signals were magnified by normalizing during- and post-signal images against the baseline mean (see percentage colorbar), followed by trial averaging (40 trials per whisker pair). Methods were based on previous studies (Aronoff and Petersen, 2007). B, For each mouse, a single blood vessel image was obtained under high-power green light (525 nm) and overlaid on the grand-average S1 barrel signal, creating a map to guide future silicon probe implantation. The signal in orange indicates >2 standard deviations from the mean. C, Examples of S1 barrel imaging from strong to dim, along with horizontally averaged cortical laminar responses (current-source density, CSD) to whisker stimulation in the awake mouse (see main manuscript, Fig. 4), as well as Nissl-stained coronal sections showing electrolytic lesion along the silicon probe tract. Download Figure 1-1, TIF file. [file eneuro-13-ENEURO.0417-25.2026-s002.tif]

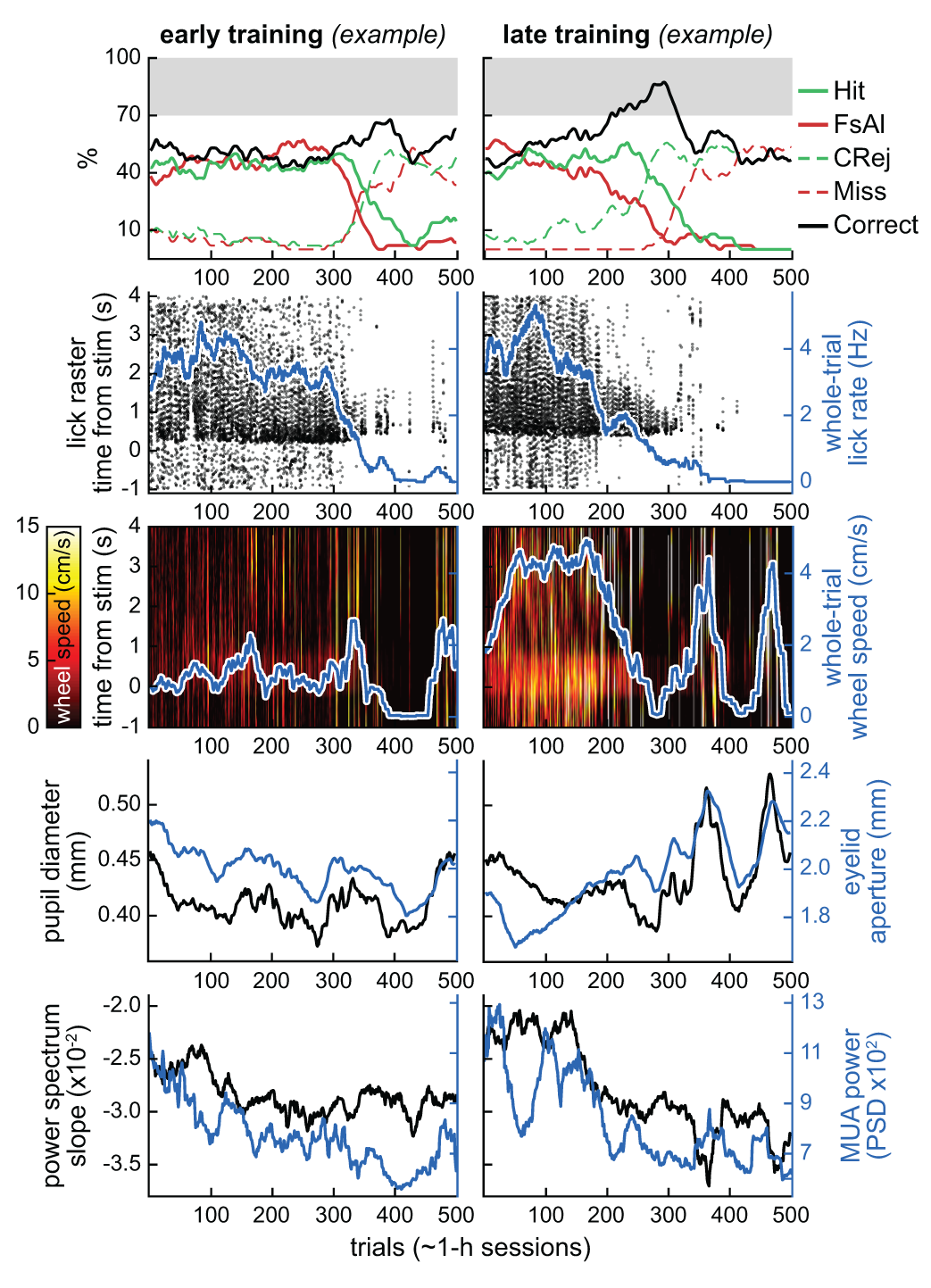

Supplement: Figure 1-2 — Qualitative illustrations from a single mouse, showing variations in behavioral and physiological metrics during two representative sessions: early and late training. All curves represent whole-trial measures (one value per trial) in chronological order (x axes), analyzed with a 50-trial moving mean. Specifically in licking and wheel activity (second and third rows of graphs), whole-trial measures (right-side y axes, blue curves) are overlaid on raster plots and heatmaps displaying peri-stimulus patterns (left-side y axes). Both longer-term trends and smaller fluctuations can be observed across graphs, with varying levels of redundancy among variables. These inter-variable relationships were examined using linear classification in the main manuscript (Fig. 2). Download Figure 1-2, TIF file. [file eneuro-13-ENEURO.0417-25.2026-s003.tif]

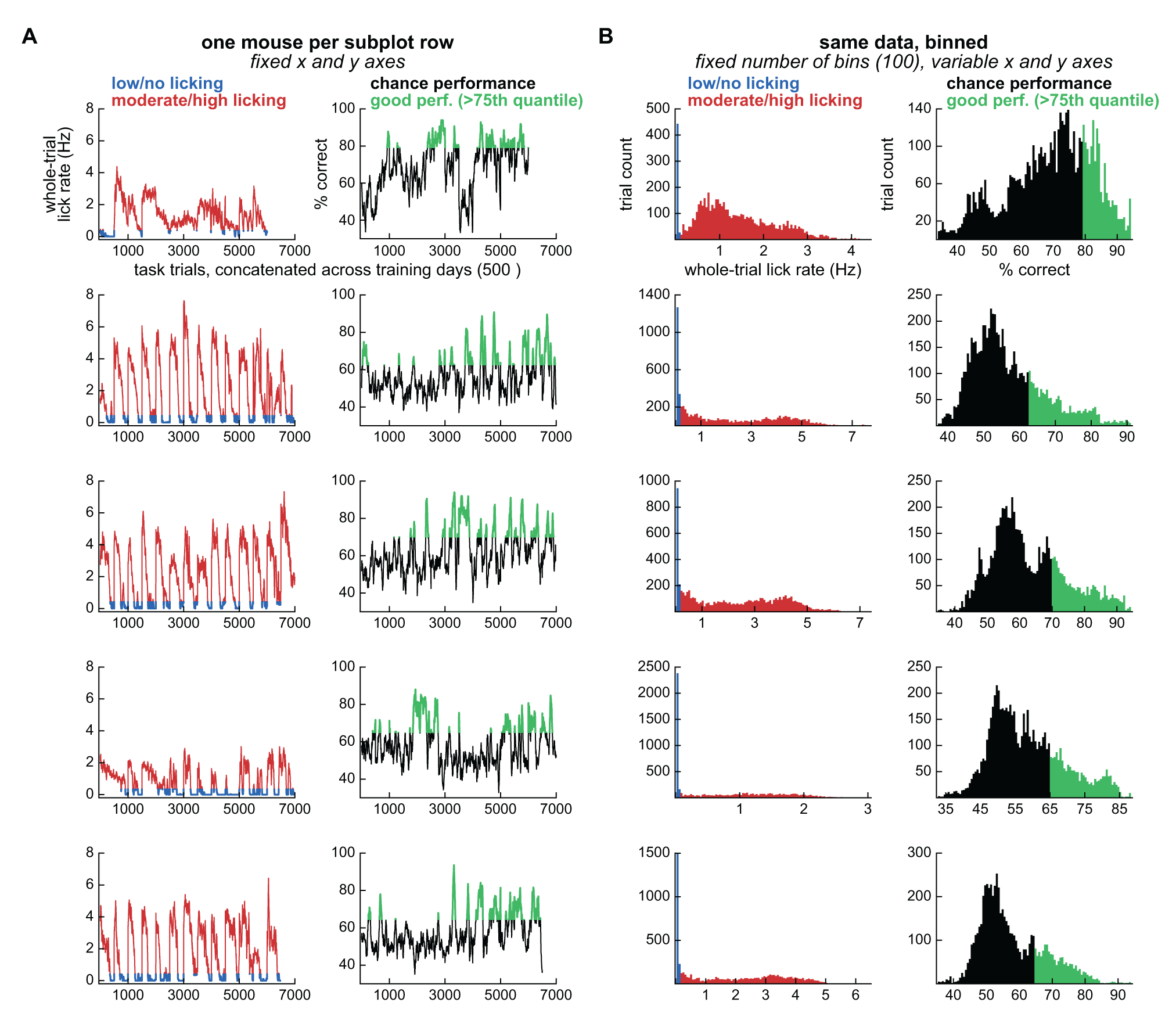

Supplement: Figure 2-1 — Licking and task correctness curves and histograms from all mice (supplementing Figs. 1F and 2A,B). A, Trials were concatenated across days (500 trials/day), illustrating variations within and between sessions. Each session was independently smoothed using a 50-trial moving mean. Color codes indicate distinct trial categories for lick rates and correctness, which were later combined into the tripartite state category system used in the main analysis (Fig. 2C). B, The same data and color coding are shown as trial count histograms, each with a fixed number of bins (100) and variable bin edges. See Methods for rationale regarding lick rate and correctness cutoffs. Download Figure 2-1, TIF file. [file eneuro-13-ENEURO.0417-25.2026-s004.tif]

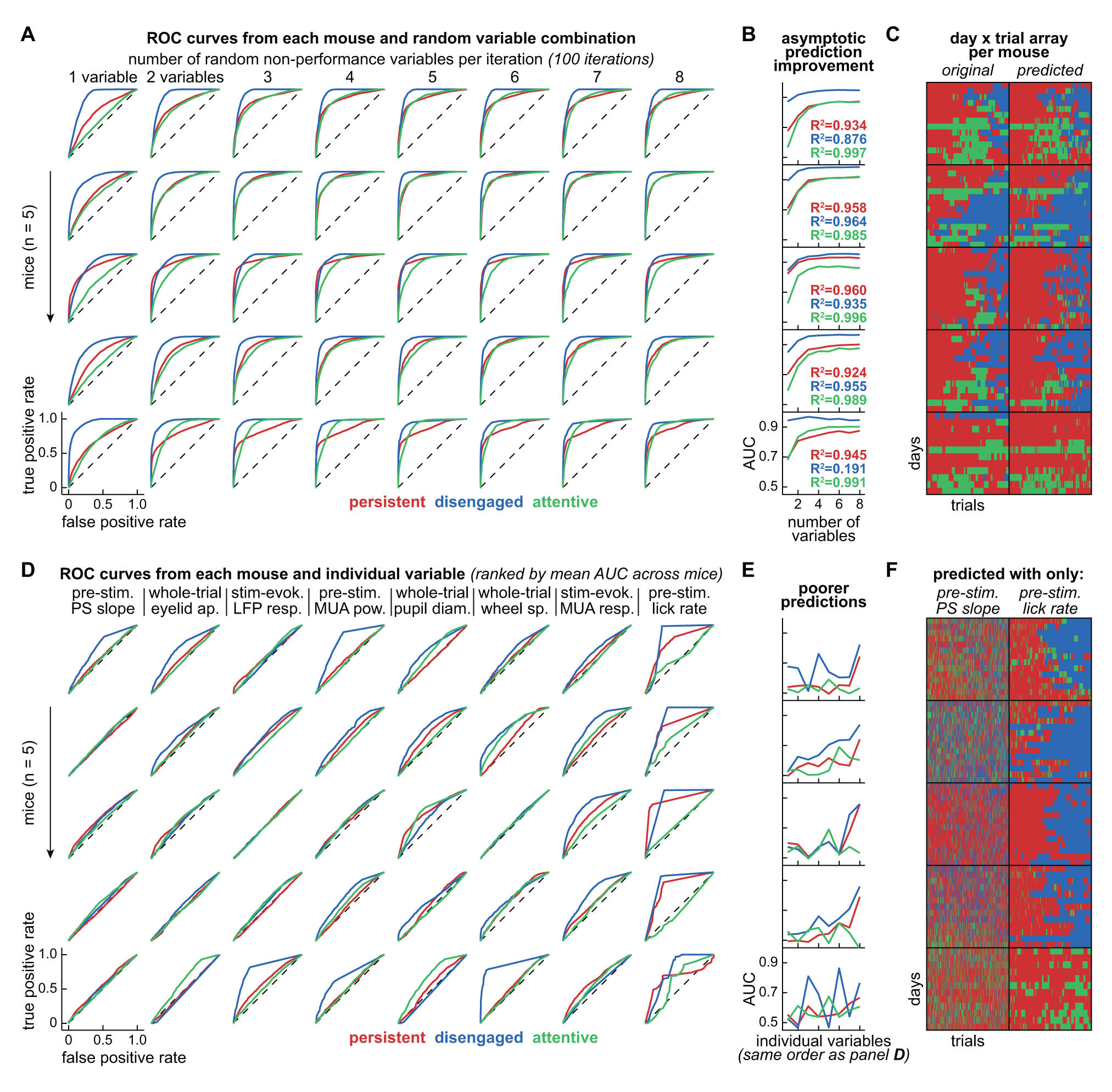

Supplement: Figure 2-2 — Supplementary individual-mouse illustrations reinforcing that performance and non-performance metrics covary with learning and arousal. A, Same receiver-operating characteristic (ROC) analysis as in the main manuscript (Fig. 2F) but depicting all mice in the rows of graphs. Linear classification of arousal states was conducted in eight groups of 100 iterations, each group using a different number of randomly-chosen non-performance predictors. Cumulative true positive and false positive rates per state were then calculated per group of 100 iterations, showing that the more diverse the pool of non-performance predictors, the better the state classification. B, Area under the curve (AUC) analysis from the same ROC data, showing asymptotic prediction improvement with increasing numbers of non-performance predictors. Prediction improvement was generally more evident for the impulsive and attentive state categories (see R-squared correlations). C, Day-by-trial state category matrices per mouse, for qualitative comparison between original and predicted categories. In this case, predictions were obtained from all eight non-performance variables. D-F, Similar layout, but using single-variable iterations, ranked by mean AUC across states. No single variable matched the classification performance obtained using multiple variables. Download Figure 2-2, TIF file. [file eneuro-13-ENEURO.0417-25.2026-s005.tif]

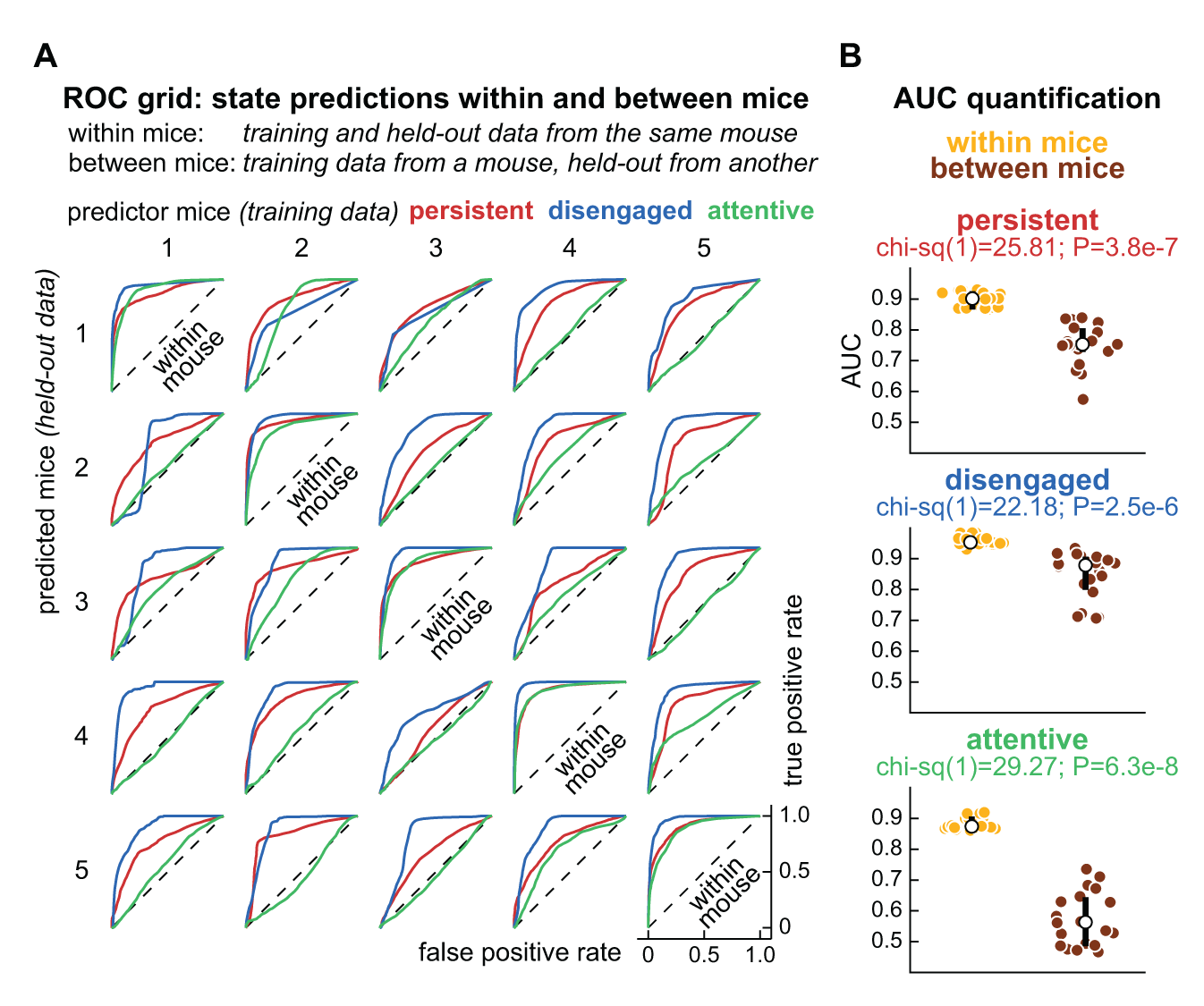

Supplement: Figure 2-3 — State predictions within each individual mouse and across all possible mouse pairs. A, Grid of receiver operating characteristic (ROC) subplots. All ROC curves were generated using the full set of eight non-performance variables. The diagonal shows within-mouse analysis, where both training and held-out data are from the same mouse. Off-diagonal subplots represent “predictor-predicted” mouse pairs, with training data from one mouse and held-out data from another (see Methods). B, AUC values from the ROC subplots in panel A, showing consistently higher predictive accuracy for individualized (within-mouse) state predictions compared to cross-mouse predictions, especially for attentive states. Download Figure 2-3, TIF file. [file eneuro-13-ENEURO.0417-25.2026-s006.tif]

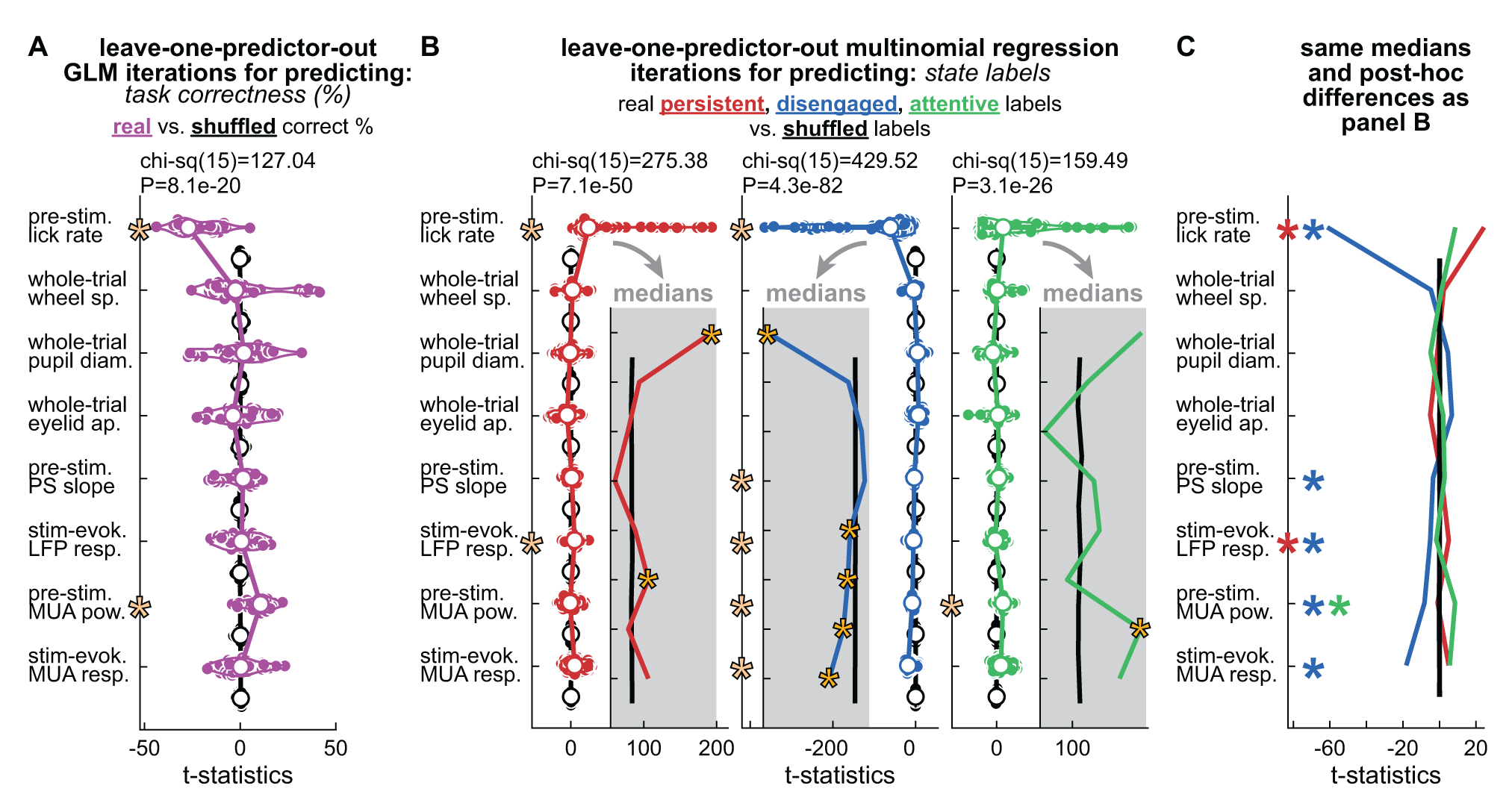

Supplement: Figure 2-4 — Alternative analysis of motivation states using Generalized Linear Models (GLM) and Multinomial Regression (MNR). A, Violin distributions of GLM t-statistic values (filled datapoints) and their medians (white datapoints) per predictor variable (y-axis). These t-statistic values were cumulatively plotted across mice, each mouse represented by multiple leave-one-predictor-out GLM iterations, where one predictor variable is omitted per iteration (intercept always removed). The response variable used in this analysis was task correctness percentage (see Figs. 1F and 2A,B, as well as Extended Data Fig. 2-1), thus testing the ability of these GLMs to predict task correctness independent of state categories. The same leave-one-predictor-out iterations were conducted for shuffled correctness percentage data, generating controls (black datapoints) for statistical comparisons using Friedman’s test (see chi-square and P values). Asterisks indicate post-hoc differences (P < 0.005) between real-data and shuffled-data iterations, using Tukey’s protection for pairwise comparisons. B, Similar analysis as panel A, but using leave-one-predictor-out MNR iterations for predicting the three state categories of the main study. In MNR, one category is always used as reference for the other categories. Thus, an additional iteration level was necessary for MNR, so that each of the three states was assigned as the reference category (i.e., one reference state per turn). T-statistic values resulting from these two MNR iteration levels (leave-one-predictor-out and reference categories) were parsed by motivation state, as shown in these three plots. Insets additionally show the medians of violin distributions, magnifying subtle differences between real-data and shuffled-data iterations. These MNR results help “decompose” the GLM patterns observed in panel A. For instance, negative t-statistic values for pre-stimulus licking and positive values for pre-stimulus MUA power in panel A can be attrib [file eneuro-13-ENEURO.0417-25.2026-s007.tif]

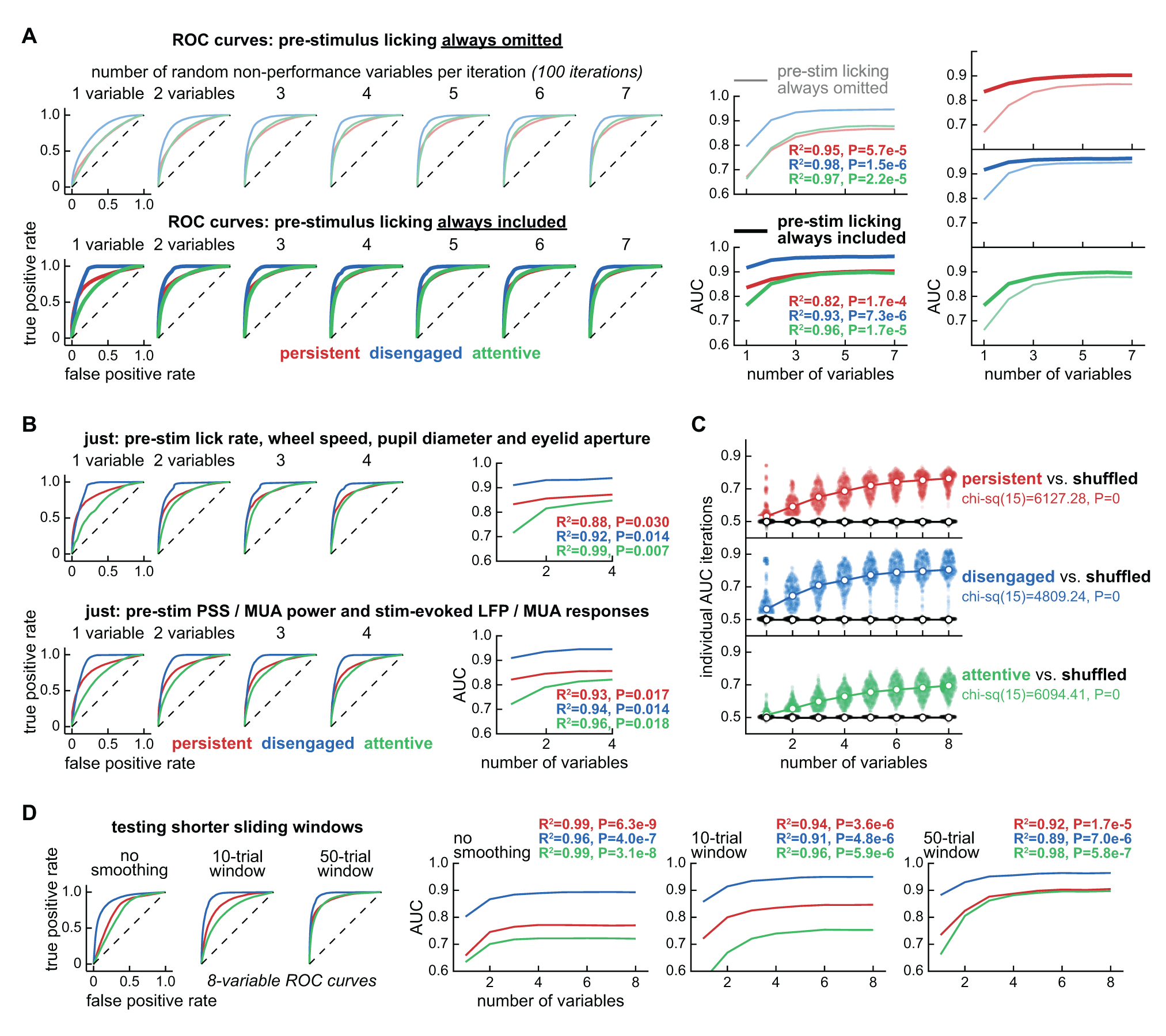

Supplement: Figure 2-5 — Several additional controls for the receiver operating characteristic (ROC) area under the curve (AUC) analysis in Fig. 2. A, Pre-stimulus licking was identified as the most influential predictor variable in Fig. 2I,J. Here, its impact on predictive accuracy was further tested through ROC-AUC iterations that either always omitted or always included this variable. When pre-stimulus licking was always omitted, the AUC curve asymptotes remained strong, but their plateaus decreased slightly, reaching 0.86 AUC for the persistent/attentive states compared to 0.89 AUC in the main analysis (Fig. 2H). Conversely, when pre-stimulus licking was always included, the asymptotic trends became weaker, and AUC values plateaued at the same level as the main analysis (Fig. 2H). Importantly, the attentive state remained the most challenging to predict in this supplementary analysis (see also Fig. 2I,J). These observations clarify that pre-stimulus licking showed partial reliability at predicting persistent and disengaged states, and insufficient reliability at predicting attentive states. B, Same ROC AUC analysis, but using two subsets of predictors: physiological/behavioral (pre-stimulus lick rates, and whole-trial wheel speed, pupil diameter and eyelid aperture) and neurophysiological (pre-stimulus PSS / MUA power, and channel-averaged stimulus-evoked LFP / MUA responses). Results revealed a modest difference between subsets; for the attentive state, the most difficult to predict, the physiology/behavior subset plateaued at 0.84 AUC versus 0.82 AUC for the neurophysiology subset. Asymptotic trends showed P values < 0.03 in all cases, though these values are interpreted with caution due to the limited number of datapoints per asymptote (four compared to eight in the main analysis in Fig. 2). This reinforces that combinations of four or more variables – whether behavioral, physiological, or neurophysiological – can better predict learning and intra-task states in this and potentially [file eneuro-13-ENEURO.0417-25.2026-s008.tif]

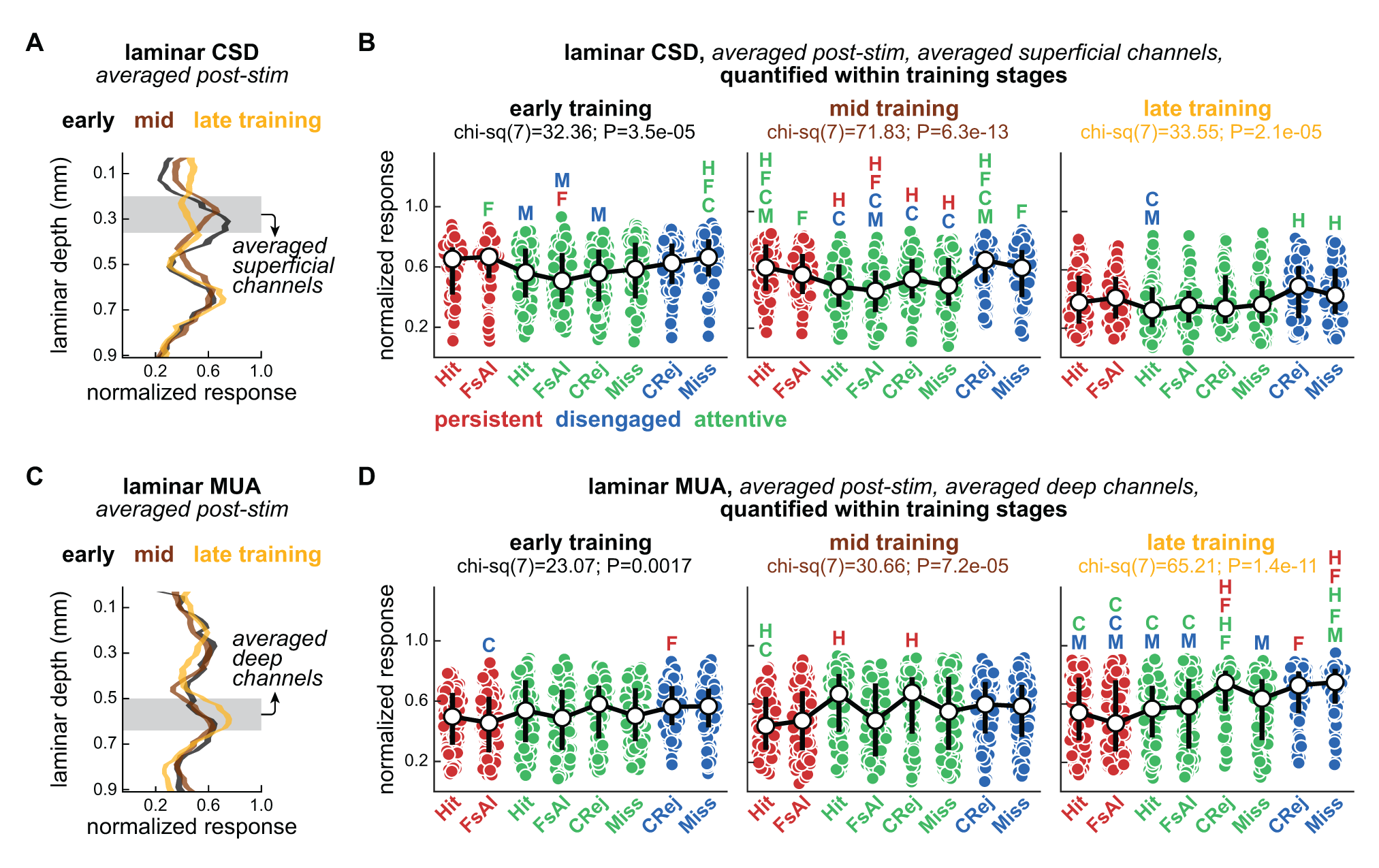

Supplement: Figure 4-1 — S1 cortex responses to whisker stimuli: specification to sensitive laminar depths, followed by state comparisons within training stages. A, Current-source density (CSD) response strengths at superficial channels (0.20-0.36 mm from brain surface) were averaged and sorted by training stage, motivation state and go/no-go outcome across mice. By removing the laminar depth dimension, this approach facilitated direct comparisons among trial types within each training stage (see Fig. 4C methods for trial sorting details). B, Sorted trials across three mice (datapoints) are shown as violin distributions, statistically quantified using Friedman’s test (see chi-square and P values). Lettered labels for go/no-go outcomes (H: hits, F: false alarms, C: correct rejections, M: misses) are color-coded by state, indicating pairwise post-hoc differences between trial types (P < 0.01, Tukey’s method). C-D, Same as panels A-B, but for multi-unit activity (MUA) response strengths at deeper channels (0.50-0.64 mm). Statistical effects varied with both electrophysiological metric and training stage: stronger for superficial CSD during mid training, and stronger for deep MUA during late training. Furthermore, post-hoc comparisons show, for example, stronger superficial CSD during early-training disengaged misses and stronger deep MUA during late-training attentive correct rejections. This supplement underscores that training stage, motivation state, and go/no-go outcome interact to modulate laminar activity profiles, as mentioned in the main text. Download Figure 4-1, TIF file. [file eneuro-13-ENEURO.0417-25.2026-s009.tif]
